# Heuristic value-based framework for lung cancer decision-making

**DOI:** 10.18632/oncotarget.25643

**Published:** 2018-07-06

**Authors:** Isa Mambetsariev, Rebecca Pharaon, Arin Nam, Kevin Knopf, Benjamin Djulbegovic, Victoria M. Villaflor, Everett E. Vokes, Ravi Salgia

**Affiliations:** ^1^ Department of Medical Oncology and Therapeutics Research, City of Hope, Duarte, CA, USA; ^2^ California Pacific Medical Center Research Institute, San Francisco, CA, USA; ^3^ Department of Clinical Supportive Care, City of Hope, Duarte, CA, USA; ^4^ Department of Medicine (Hematology and Oncology), Northwestern University, Chicago, IL, USA; ^5^ Department of Medicine, University of Chicago, Chicago, IL, USA

**Keywords:** non-small cell lung cancer, heuristics, fast-and-frugal trees, genomics, framework

## Abstract

Heuristics and the application of fast-and-frugal trees may play a role in establishing a clinical decision-making framework for value-based oncology. We determined whether clinical decision-making in oncology can be structured heuristically based on the timeline of the patient's treatment, clinical intuition, and evidence-based medicine. A group of 20 patients with advanced non-small cell lung cancer (NSCLC) were enrolled into the study for extensive treatment analysis and sequential decision-making. The extensive clinical and genomic data allowed us to evaluate the methodology and efficacy of fast-and-frugal trees as a way to quantify clinical decision-making. The results of the small cohort will be used to further advance the heuristic framework as a way of evaluating a large number of patients within registries. Among the cohort whose data was analyzed, substitution and amplification mutations occurred most frequently. The top five most prevalent genomic alterations were TP53 (45%), ALK (40%), LRP1B (30%), CDKN2A (25%), and MYC (25%). These 20 cases were analyzed by this clinical decision-making process and separated into two distinctions: 10 straightforward cases that represented a clearer decision-making path and 10 complex cases that represented a more intricate treatment pathway. The myriad of information from each case and their distinct pathways was applied to create the foundation of a framework for lung cancer decision-making as an aid for oncologists. In late-stage lung cancer patients, the fast-and-frugal heuristics can be utilized as a strategy of quantifying proper decision-making with limited information.

## INTRODUCTION

Lung cancer, the leading cause of cancer death among all ages, is expected to account for 234,030 newly diagnosed cases and 154,050 cancer related deaths in the United States in 2018 [1]. Lung cancer is histologically and molecularly heterogeneous with several subtypes and molecular alterations present. Non-small cell lung carcinoma (NSCLC) is one of the four major histologic types of lung cancer and represents approximately 85% of all lung cancer [2]. NSCLC is comprised of three main subtypes: adenocarcinoma, squamous cell carcinoma, and large cell carcinoma. Adenocarcinoma is the most common subtype of NSCLC, comprising approximately 40% to 50% of all lung cancer [3, 4, 5].

While years ago we relied solely on histopathology for information, the newer science of genomic-based classification of NSCLC has blurred the lines among histologic subtypes by demonstrating that lung cancer can be molecularly subclassified as either adenocarcinoma or neuroendocrine tumor based on its molecular markers [6]. This development of genomic-based characterization has shifted the focus of lung cancer treatment from merely general cytotoxic chemotherapy and radiation treatments to an integration of newer targeted therapeutics that can overcome the challenges of chemotherapeutic resistance and disease progression [7]. The discovery of various genetic driver mutations such as gain-of-function EGFR and KRAS mutations, ALK and ROS1 translocations, and MET amplifications/mutations has vastly improved the basic understanding of the biology of NSCLC [8]. This has also allowed for the development of targeted therapies for these mutations, such as EGFR tyrosine-kinase inhibitors which include erlotinib, gefitinib and afatinib, as well as newer third-generation TKIs such as osimertinib [9]. This new approach of genomic-based classification presents a challenge not only biologically but also clinically as more kinase inhibitors are developed and the biological understanding of driver mutations and NSCLC is further accentuated.

The clinical scenarios are more complex than just genomic heterogeneity. Although NSCLC is driven by multiple genetic pathways, no two cancers are alike, just as no two patients are alike in their treatments or philosophy of care. Integrating disparate information—clinical, biological, and psychological—remains the art of medicine. To improvise, one must have comfort with uncertainty and the basics of decision analysis in real time. A contemporary oncologist is tasked with constantly making tradeoffs in the clinic based on imprecise information, also known as improvising. We propose an idea of a heuristic framework for clinical decision-making, using key information from each patient's diagnosis, genomics, and cancer information that will allow physicians to make accurate decisions regarding the treatment plans of their patients. We describe below clinical scenarios from patients with NSCLC, arbitrarily divided into 10 “straightforward” cases and 10 “complex” cases by the degree of clinical intuition and complexity required to reach an optimal solution Supplementary Figures 1-20. The cases serve as a small template for a broader heuristic evaluation utilizing fast-and-frugal trees to understand the complexity of clinical care and accurate decision-making.

## RESULTS

**Figure 1:**
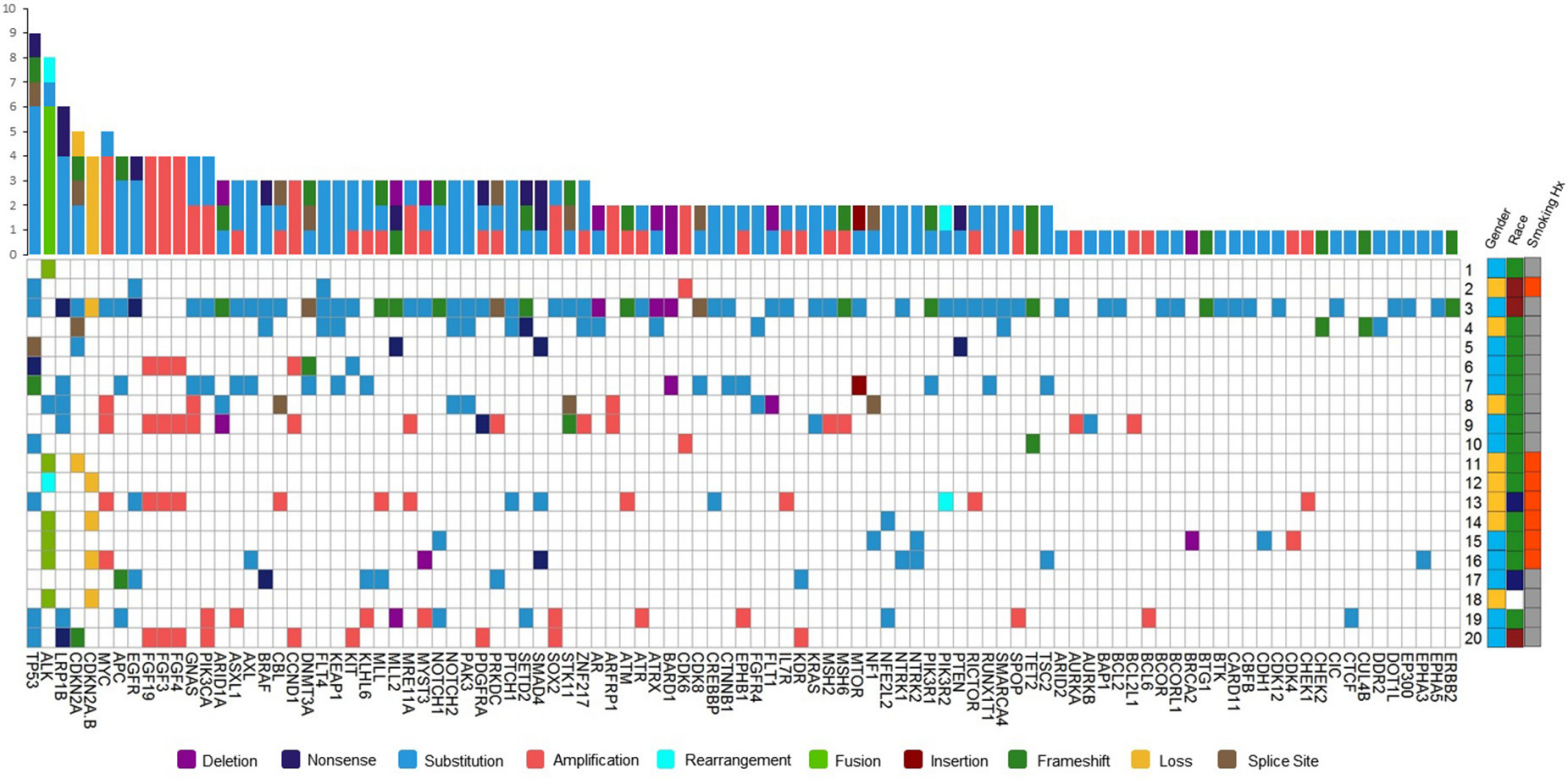
Mutational profile of 20 patients represented by a heatmap (left) The most frequent genomic alterations identified in this cohort are displayed on the left side of the heatmap. Overall, the most common types of mutations in this cohort were substitution and amplification shown by the mutational frequency bar plot above the heatmap. The heatmap on the right represents the gender, smoking history, and race of the cohort (Gender: Male = blue, Female = yellow; Smoking History: Nonsmoker = orange, Smoker = gray; Race: Caucasian = green, African American = red, Asian = dark blue, Undisclosed = white).

**Figure 2:**
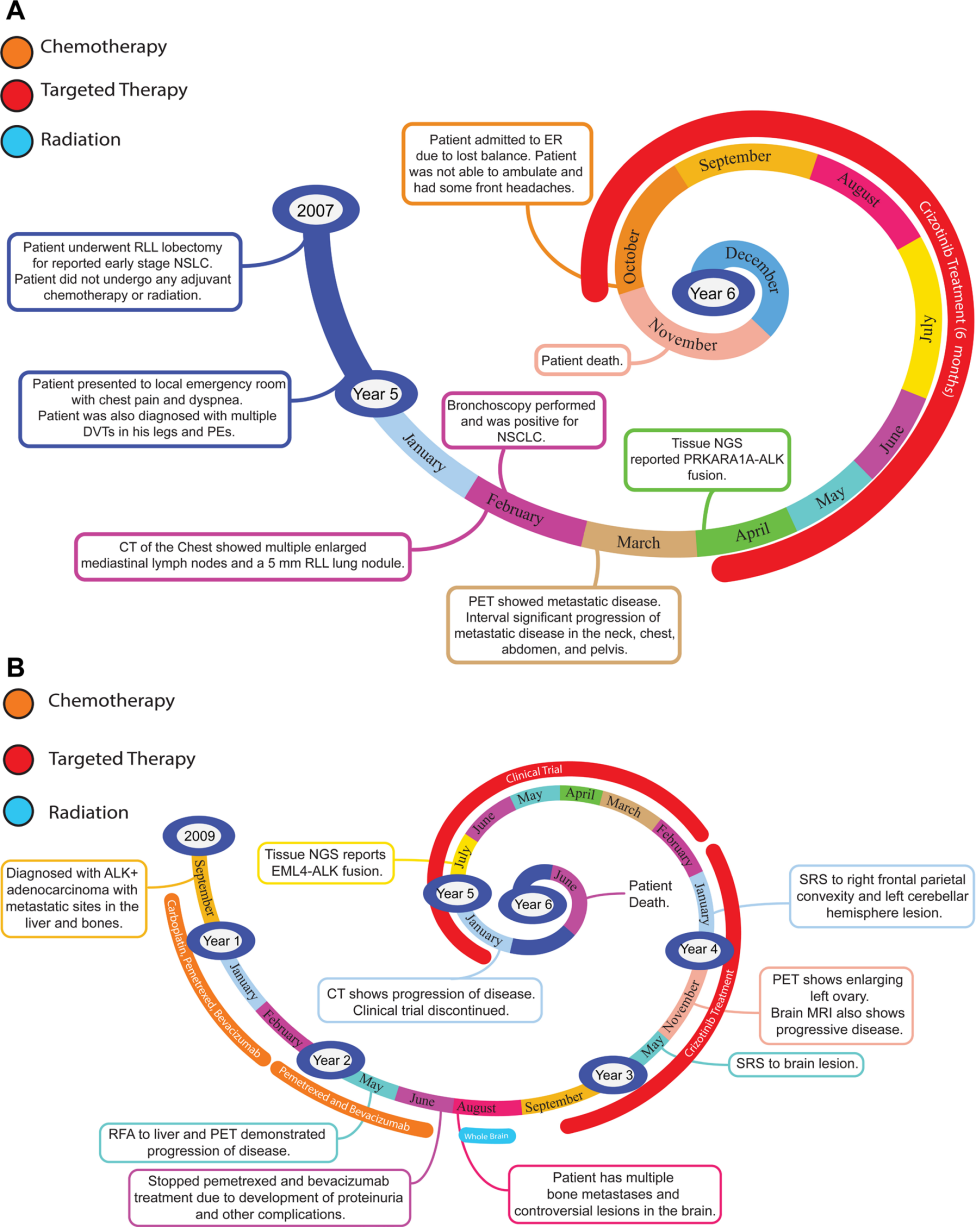
Representative timelines of patient treatment history (**A**) Timeline representation of straightforward case #1 with ALK positive NSCLC which did not undergo any adjuvant chemotherapy or radiation and eventually progressed with diffuse metastatic disease 5 years after diagnosis. (**B**) Timeline representation of complex case #4 with ALK positive adenocarcinoma that presented with extensive metastatic disease but was well managed and survived for an additional 5 years after diagnosis.

**Figure 3:**
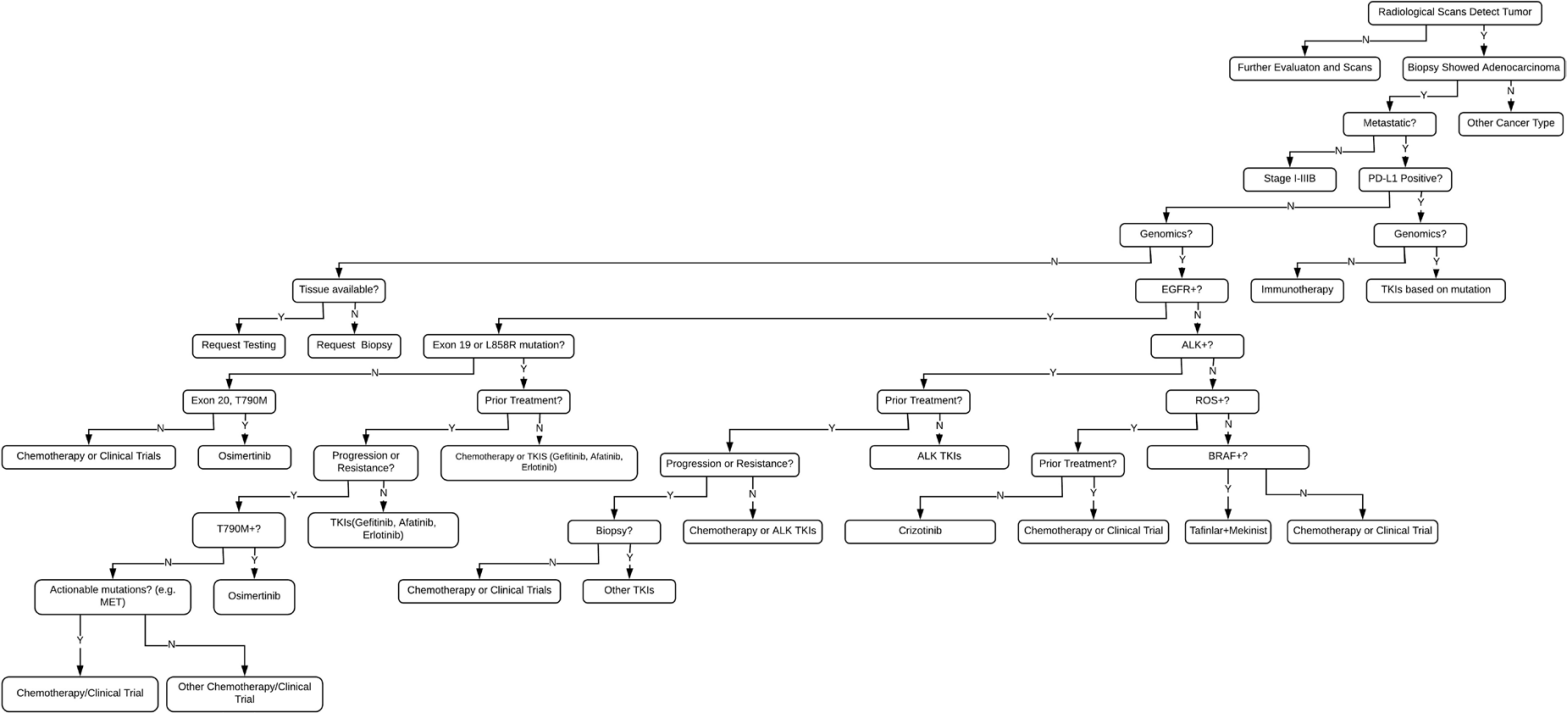
Genomic FFT tree for actionable mutations in NSCLC

**Figure 4:**
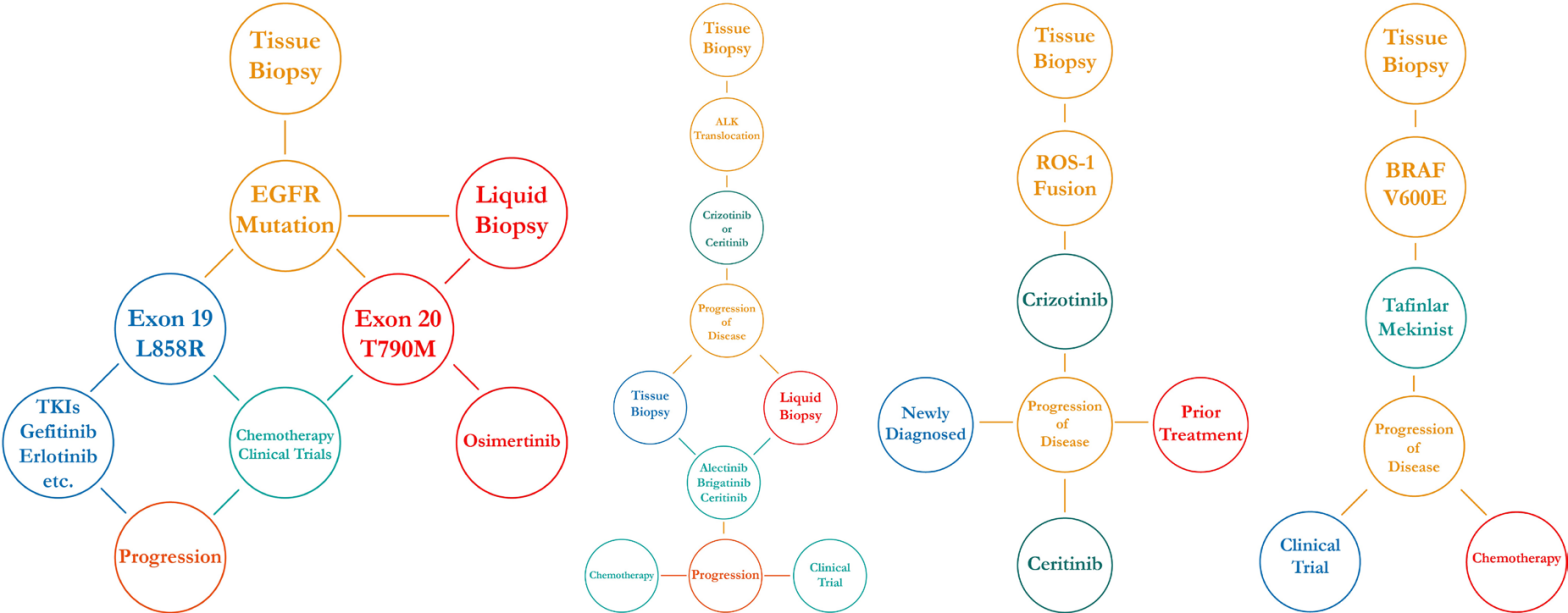
Dendograms of clinical decision-making for actionable mutations in NSCLC

There were ten types of mutations represented in this cohort and the majority of patients had more than one genetic mutation reported. After the genetic reports were compiled, the genes were sorted by frequency and correlated to the de-identified patient number as shown in a mutational tile plot (Figure 1). Mutational frequency is represented by the bar plot as well. Overall, the most common types of mutations found in this cohort were substitution and amplification. Genomic alterations were most frequently found in *TP53* (9 out of 20 patients, 45%), *ALK* (8 out of 20, 40%), *LRP1B* (6 out of 20, 30%), *CDKN2A* (5 out of 20, 25%), and *MYC* (5 out of 20, 25%). Demographic information was also assessed as presented in the tile plot by gender, smoking history, and race. Among the cohort, majority of patients were male (12 patients, 60%) and had a history of smoking (13 patients, 65%). The most prevalent race was Caucasian (14 patients, 70%), with the rest identifying themselves as African American, Asian, or undisclosed. In Table 1 and Table 2 we evaluated the clinical decision-making process for 10 cases that were deemed straightforward and 10 cases that were deemed complex based on the number of steps taken during their treatment. Figure 2 shows a patient timeline of treatment for a straightforward and a complex case. Figure 3 and Figure 4 represent the initial steps towards creating a framework for lung cancer decision-making that would help guide physicians in patient treatment plans.

**Table 1:** Straightforward cases

Structure	Case Summary
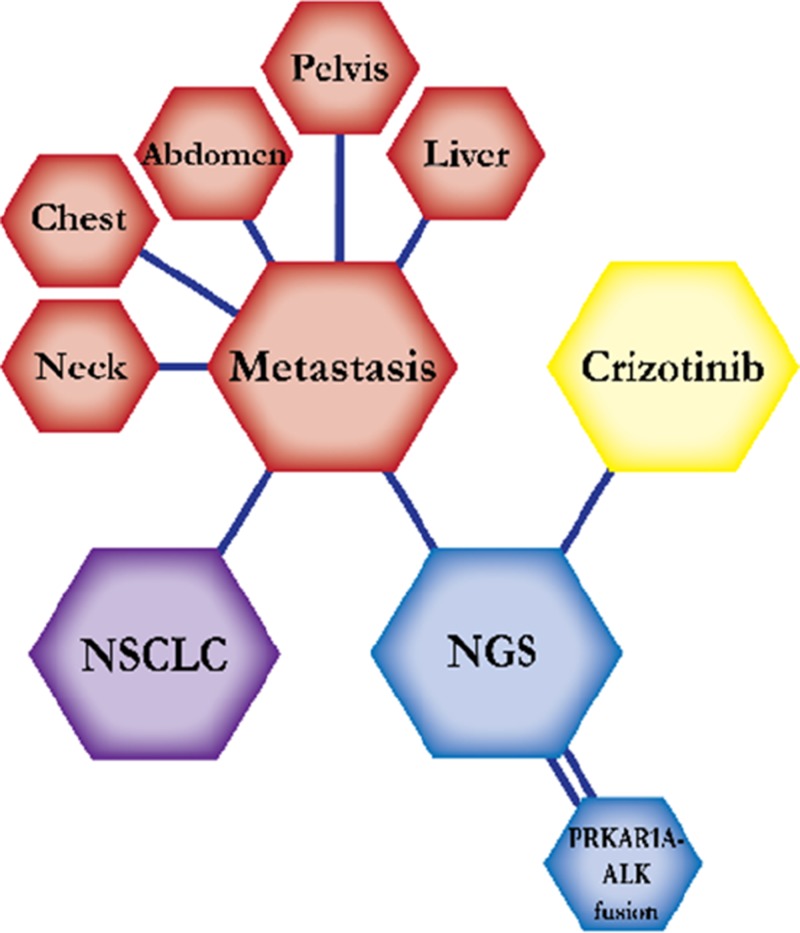	A 68-year-old Caucasian male smoker with a history of 30 pack years was diagnosed with early stage non-small cell carcinoma, monitored with follow-up scans for five years. The patient then presented to the ER with chest pain and dyspnea. Workup showed metastatic disease with progression in the neck, chest, abdomen, liver, and pelvis. He was planned to start on palliative chemotherapy, which is not expected to prolong survival but improve quality of life. With this treatment the patient was expected to live two months. However, molecular testing by NGS showed a PRKARA1A-ALK fusion. The patient was then immediately started on a regimen of crizotinib at a dose of 250 mg per day and showed clinical and radiological response. Crizotinib is effective for patients with ALK-positive mutations and inhibits oncogenic activity of the kinase. He was expected to prolong progression-free survival for about seven months on this treatment regimen. The patient continued on crizotinib for six months before he was admitted to the ER due to loss of balance and passed away.
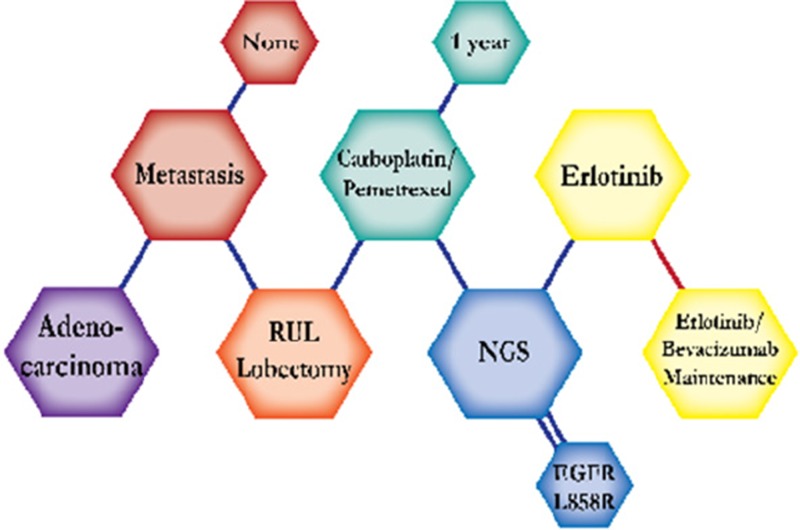	A 70-year-old African American female non-smoker presented with a RUL mass, positive for adenocarcinoma. She underwent a RUL lobectomy and started on carboplatin and pemetrexed. Combination chemotherapy of Carboplatin and Pemetrexed is expected to prolong survival by four months compared to pemetrexed alone. However, results from a NGS test revealed an EGFR L858R exon 21 mutation of EGFR. Therefore, the decision was made to switch her treatment to erlotinib. Erlotinib is effective for patients with EGFR-positive mutations and inhibits its oncogenic activity. She continued on erlotinib for five months until a CT scan revealed an increase in nodules. She was switched to maintenance bevacizumab in addition to her erlotinib to inhibit angiogenesis via anti-VEGF activity. This addition to her regimen is expected to slow progression and she continues treatment.
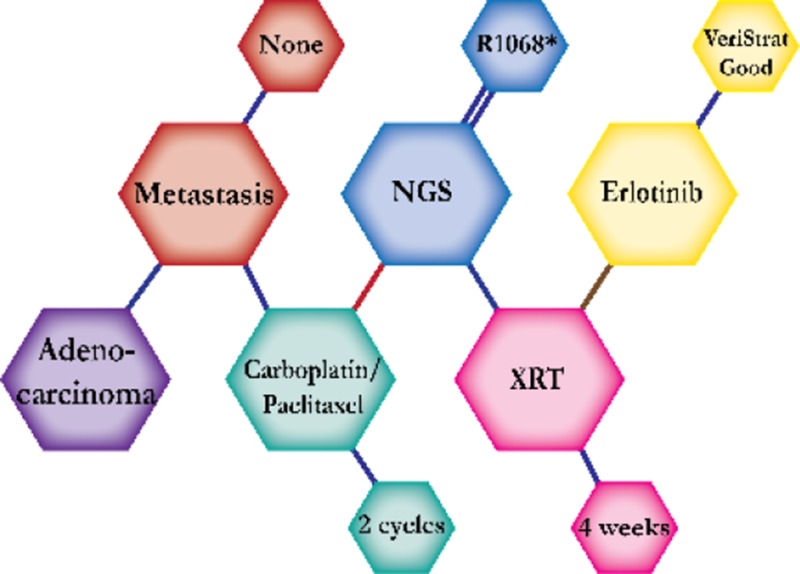	A 66-year-old African American male smoker was initially diagnosed with adenocarcinoma. A PET scan revealed a hypermetabolic tumor in the anterior mediastinum, right supraclavicular, and right axillary lymph node. The patient started treatment with Carboplatin and Paclitaxel, which was expected to improve his one-year survival by approximately 50 percent. Results from NGS reported an R1068* EGFR mutation, but no actionable mutations. He was then given 60 Gy of concurrent radiation therapy. Patient was tested for predictive prognostic response to erlotinib through a VeriStrat test and the results were VeriStrat Good. Therefore, the decision was made to start erlotinib treatment, which inhibits the mutated receptor. The patient continued on erlotinib with minimal symptoms and is expected to prolong survival by 19 percent.
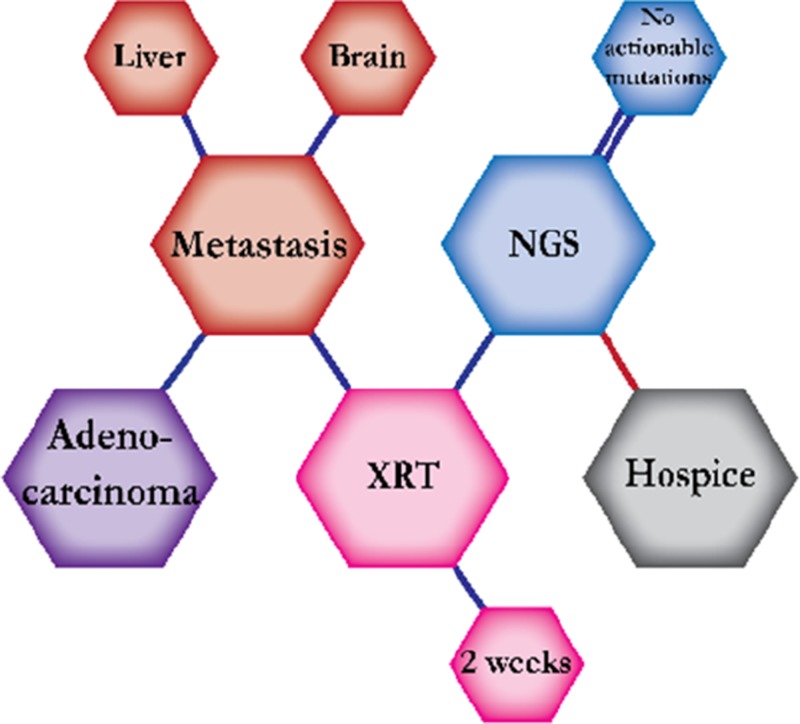	A 62-year-old Caucasian female smoker was symptomatic, but ignored her symptoms until a PET scan revealed LUL mass, positive for adenocarcinoma. A subsequent brain MRI was positive for metastasis. She started radiation therapy of 30 Gy to the LUL for two weeks to shrink the tumor. The patient then underwent NGS testing but it reported no actionable mutations. Therefore, chemotherapy was planned as the main course of treatment. However, the disease progressed rapidly and the patient was moved to hospice.
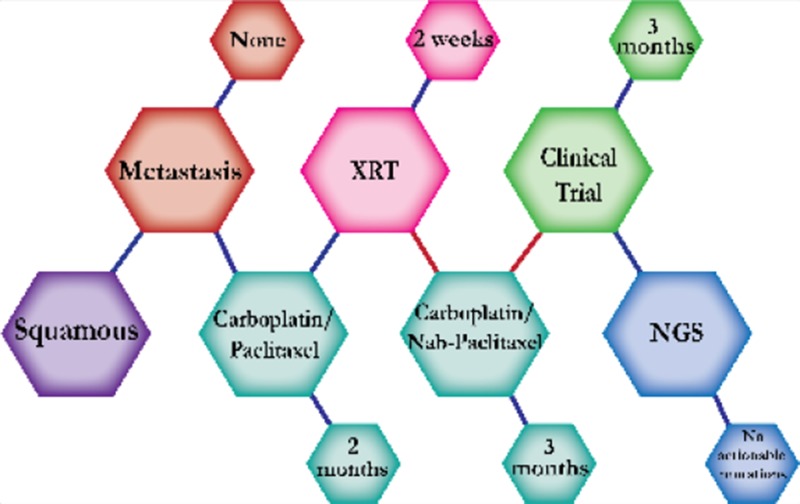	A 72-year-old Caucasian male smoker was first evaluated for cough in 2012 and workup showed squamous cell carcinoma of the RUL. He started concurrent chemotherapy (carboplatin/paclitaxel) with radiation. Patients are expected to respond better to this combined-modality regimen than either treatment alone. Upon progression of disease, treatment was switched to carboplatin/nab-paclitaxel. NGS identified no actionable mutations and the patient started on a clinical trial.
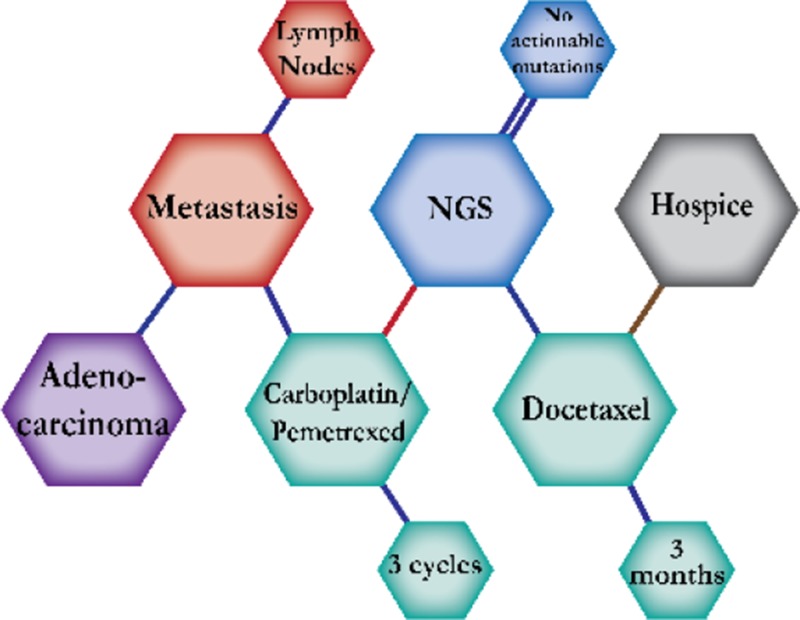	A 68-year-old Caucasian male smoker was diagnosed with metastatic adenocarcinoma. He started on carboplatin and pemetrexed. Combination chemotherapy of carboplatin and pemetrexed is expected to prolong survival by four months compared to pemetrexed alone. NGS testing reported a KRAS G12A alteration but no actionable mutations. Therefore, he continued on chemotherapy until a CT and PET scan showed progression. His treatment was switched to docetaxel and he stayed on treatment for two months. Second-line docetaxel for patients previously treated with platinum-based chemotherapy is expected to prolong survival by three months. However, docetaxel was held due to toxicity. The patient progressed rapidly and agreed to hospice care.
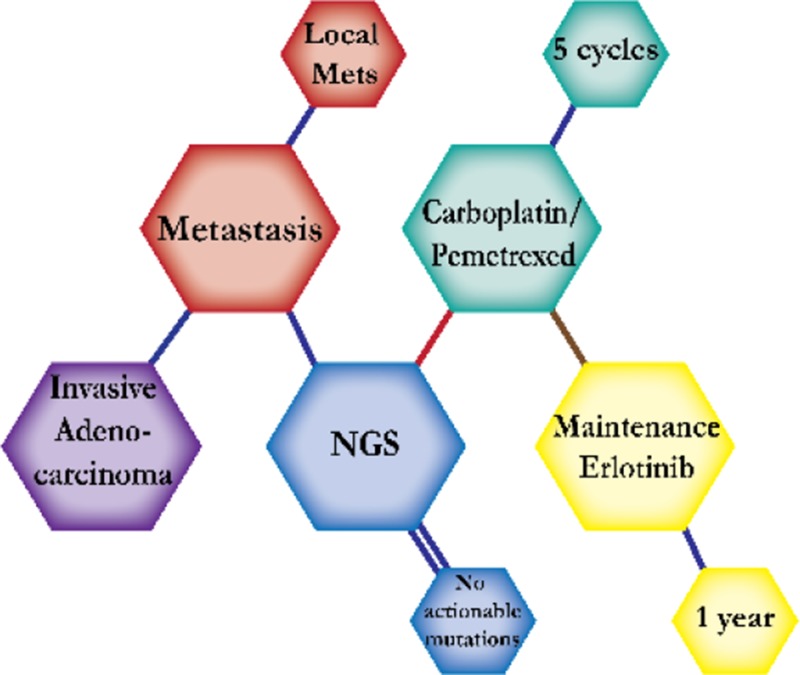	A 72-year-old Caucasian male was first presented to a hospital with neck pain. Workup showed invasive adenocarcinoma of the lung with local metastasis. The patient started on carboplatin and pemetrexed and tissue was sent for molecular testing. Combination chemotherapy of carboplatin and pemetrexed is expected to prolong survival by four months compared to pemetrexed alone. NGS testing reported no actionable mutations. He tolerated five cycles and a CT scan showed stable disease. The patient was then switched to maintenance erlotinib (150 mg). Patients with stable disease after first-line chemotherapy are expected to respond to maintenance erlotinib with prolonged overall survival by more than two months than those with complete or partial response. He tolerated erlotinib well and continues with stable disease.
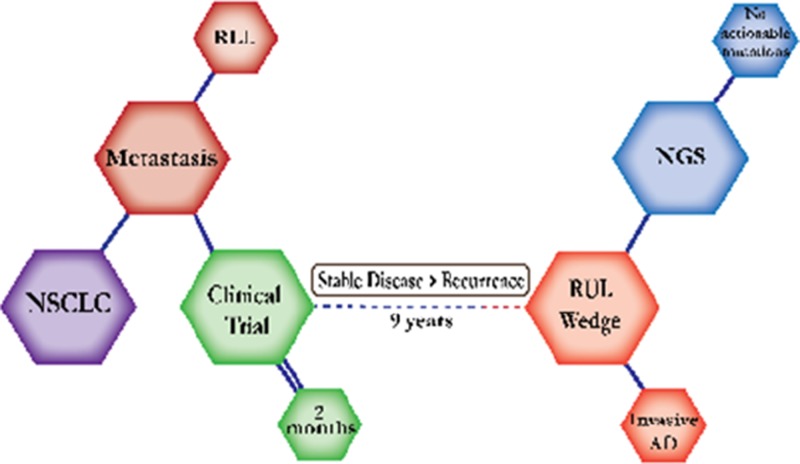	A 79-year-old Caucasian female smoker was first diagnosed with Stage IV adenocarcinoma of the RUL. She was on a clinical trial for two months and continued to do well for seven years until a PET scan showed an enlarged hypermetabolic lymph node. She was off treatment for another two months with stable scans until one scan showed considerable suspicious growth. She underwent a right upper lobe wedge resection and continued off treatment with follow-up scans every six months.
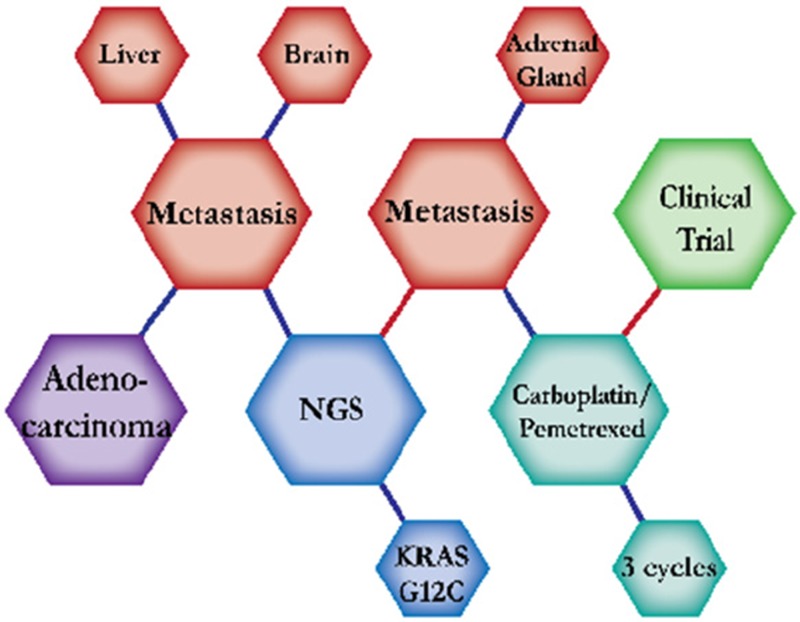	A 63-year-old Caucasian male smoker with NSCLC was initially presented with a right hilar mass. Further workup showed adenocarcinoma of RUL and metastasis in the brain and liver. NGS testing revealed no actionable mutations. Further scans revealed metastasis in the left adrenal gland. The recommended plan of care was a combination of carboplatin and pemetrexed. Combination chemotherapy of carboplatin and pemetrexed is expected to prolong survival by four months compared to pemetrexed alone. He tolerated this treatment regimen well, but further progression in the adrenal mass prompted a change of treatment and he was enrolled into a clinical trial.
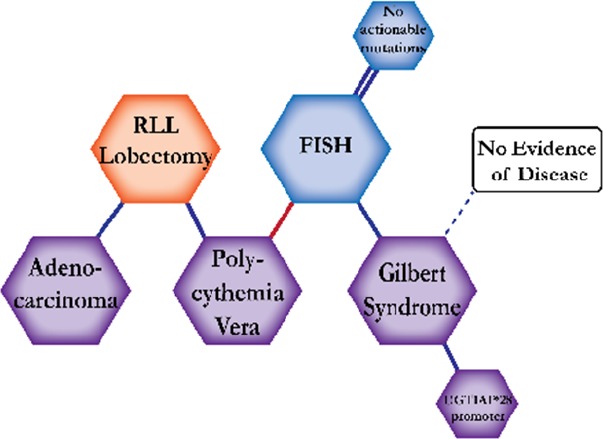	A 76-year-old Caucasian male smoker with a history of Stage IB lung adenocarcinoma. A routine CT scan showed a speculated mass in the RLL, but a PET scan revealed no FDG activity. The patient was diagnosed with polycythemia vera and testing came back positive for a V617F JAK2 mutation. UGT1A1 genotyping revealed a UGT1A1*28 promoter variant present, which supported the diagnosis of Gilbert syndrome. NGS testing revealed no actionable mutations and the patient continued off treatment with routine scans showing no evidence of disease.

The clinical structures of cases are read from left to right in chronological order.

**Table 2:** Complex cases

Structure	Case Summary
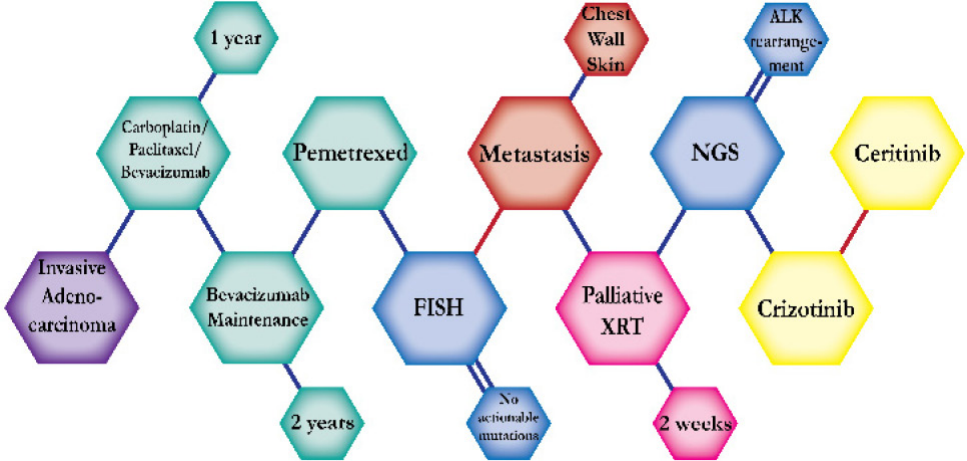	A 74 year-old Caucasian female never-smoker initially presented with invasive adenocarcinoma. She was treated with carboplatin, paclitaxel, and bevacizumab then maintenance bevacizumab with a good response. The addition of bevacizumab with the standard chemotherapy regimen is found to improve overall survival and progression-free survival in patients with advanced NSCLC. The patient was then switched to pemetrexed but stopped due to fatigue. NGS testing revealed an ALK-rearrangement and the patient started on crizotinib. Crizotinib is effective for patients with ALK-positive mutations and is expected to prolong progression-free survival for about seven months. She tolerated crizotinib with good response for sixteen months but was discontinued due to toxicities. The patient was switched to ceritinib, an ALK inhibitor targeted therapy effective for patients who progressed on crizotinib.
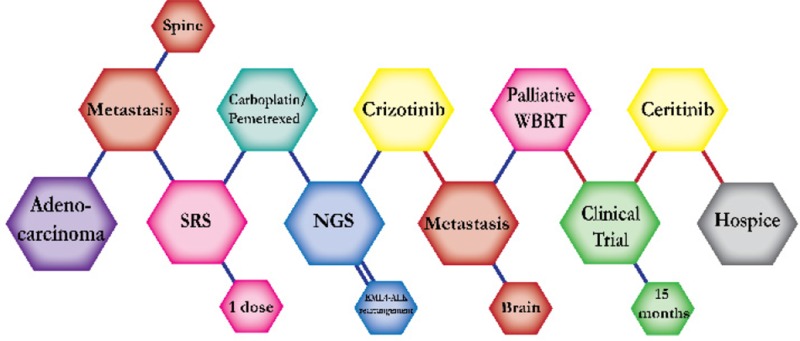	A 32 year-old Caucasian female nonsmoker who initially presented with multiple RL nodules and metastatic disease in the spine. She underwent spine radiation and started on carboplatin and pemetrexed. Combination chemotherapy of carboplatin and pemetrexed is expected to prolong survival by four months compared to pemetrexed alone. NGS testing revealed an EML4-ALK rearrangement. She was immediately started on crizotinib, a targeted therapy effective for patients with ALK-positive mutations expected to prolong progression-free survival for about seven months. She restarted crizotinib despite secondary symptoms of nausea/diarrhea until progression of disease. She received palliative whole brain radiation to treat the brain metastases. Such therapy is not expected to prolong survival but to relieve symptoms. She was then placed on a clinical trial until her disease progressed. She was switched to ceritinib, an ALK inhibitor targeted therapy effective for patients who progressed on crizotinib, but showed poor response and high toxicity. She was then transferred to hospice.
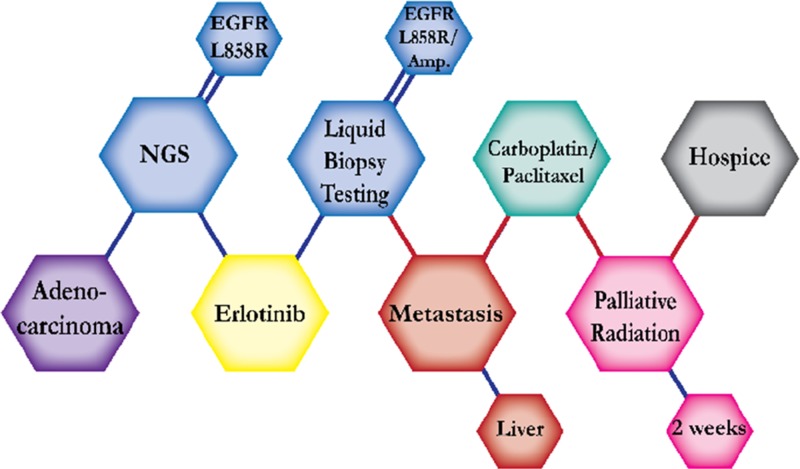	A 62 year-old Asian female nonsmoker with lung adenocarcinoma was EGFR L858R positive by NGS and started on erlotinib treatment. Erlotinib is effective for patients with EGFR-positive mutations and inhibits its oncogenic activity. A suspicious liver lesion was detected on a CT scan and a liquid biopsy was performed, but there was no T790M mutation. The appearance of an EGFR T790M mutation in liquid or tissue biopsies is a known mechanism of resistance to erlotinib. The patient then progressed by liver metastasis and was switched to carboplatin and paclitaxel. Combination chemotherapy of carboplatin and pemetrexed is expected to prolong survival by four months compared to pemetrexed alone. There was little to no response and the patient was transferred to hospice.
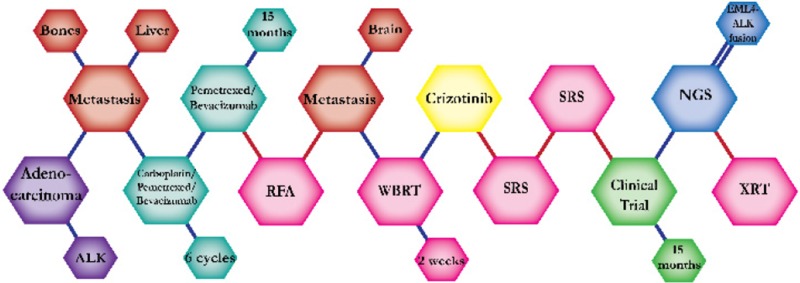	A 53 year-old Caucasian female non-smoker was diagnosed with ALK positive adenocarcinoma metastatic to the liver and bones. She started treatment with carboplatin, pemetrexed, and bevacizumab. The addition of bevacizumab with the standard chemotherapy regimen is found prolong overall survival in patients with non-squamous NSCLC. She then went on to pemetrexed and bevacizumab maintenance, known to have a significant progression-free survival advantage, but was discontinued upon proteinuria and other complications. Patient had developed suspicious brain lesions and underwent whole brain radiation. She was then put on crizotinib. Patient progressed on crizotinib and underwent SRS to mitigate the tumor with greater precision than whole brain radiation therapy. Patient was put on clinical trial for a year but developed progression. The patient died four months later after being ineligible for another trial and unresponsive to further radiation.
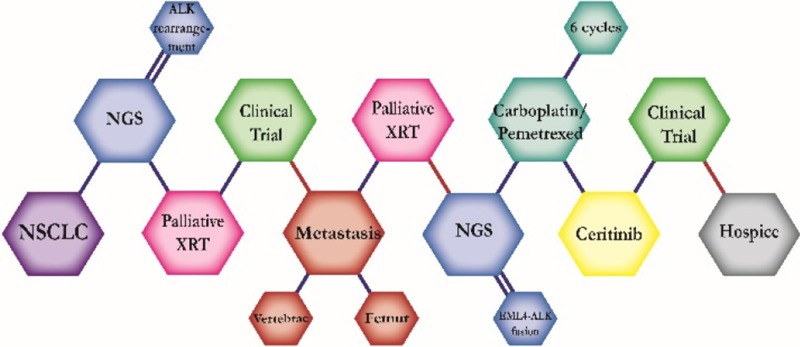	A 64 year-old Caucasian male who presented with NSCLC with an ALK rearrangement mutation. He had received one dose of palliative radiation before consultation, not mean to prolong survival but improve symptoms, and was then placed on a clinical trial. The patient continued on the trial despite progression of bone lesions due to good clinical response. However, after 12 months on treatment the patient developed further metastases in the bone. Palliative radiation therapy was done and NGS testing was done which revealed an EML4-ALK fusion. The patient began carboplatin and pemetrexed for six cycles. Combination chemotherapy of carboplatin and pemetrexed is expected to prolong survival by four months compared to pemetrexed alone. The patient started ceritinib, an ALK inhibitor, which he tolerated for 12 months until fluctuating LFTs. The patient was then put on a clinical trial. This was tolerated well until the patient developed progressive disease and was transferred to hospice.
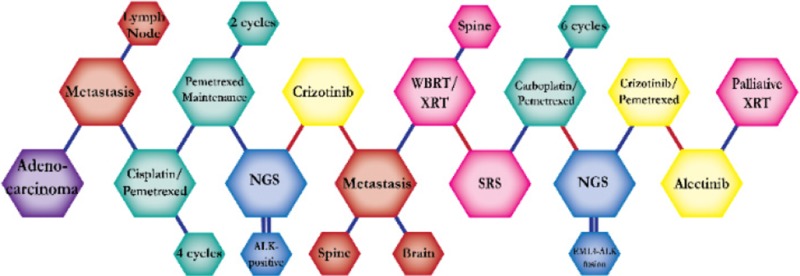	A 41 year-old Caucasian male nonsmoker who was initially diagnosed with metastatic adenocarcinoma. He started on carboplatin and pemetrexed, expected to prolong survival by four months compared to pemetrexed alone. He was offered a clinical trial but opted for continued chemotherapy with two cycles of pemetrexed maintenance, known to prolong overall survival and progression-free survival significantly. NGS revealed an ALK positive mutation and around the same time a CT confirmed progression of disease. Therefore, the patient immediately started on crizotinib, a targeted therapy effective for patients with ALK-positive mutations expected to prolong progression-free survival for about seven months. He remained on crizotinib for eight months but new lesions were detected. The patient underwent radiation therapy to the brain and spine and then was started on six cycles of carboplatin and pemetrexed. A NGS test from brain resection tissue reported EML4-ALK fusion. The patient was switched to crizotinib and pemetrexed combination maintenance, reported to have sustained clinical benefit in the CNS and minimal toxicities. This combination therapy was given for seven months until further progression. The patient was immediately switched to alectinib, an ALK inhibitor, but there was little to no response. The patient received palliative radiation therapy to the spine but it was solely to relieve symptoms and the patient died a month later.
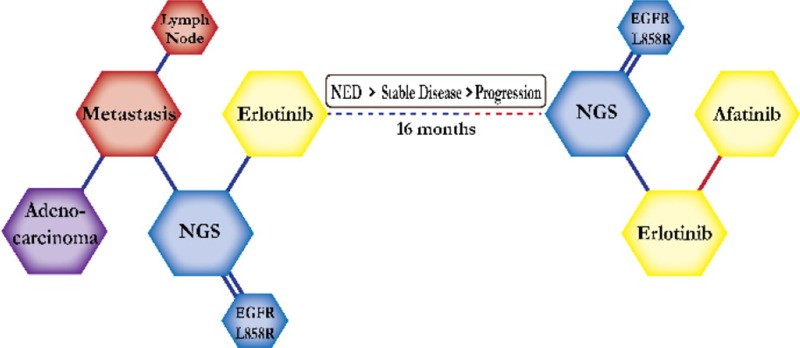	A 78 year-old Asian male smoker who was first diagnosed with a LUL mass positive for adenocarcinoma. A surgical resection was attempted but was deemed inoperable due to metastatic lymph nodes. NGS testing revealed an EGFR L858R mutation. Therefore, the patient was started on erlotinib, an EGFR inhibitor that increases progression-free survival in patients. He tolerated erlotinib for fifteen months until he was hospitalized for pleural effusion. The patient was immediately switched to afatinib, an EGFR inhibitor that has efficacy as a treatment after erlotinib progression, but showed toxicities and little to no response. The patient died in three months.
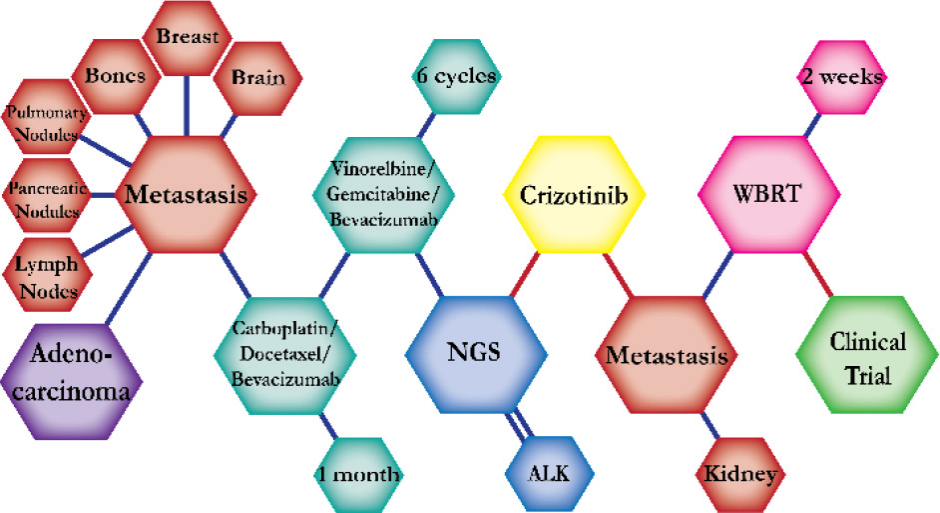	A 53 year-old female smoker who was first diagnosed with extensive metastatic adenocarcinoma. The patient was started on aggressive chemotherapy of carboplatin, docetaxel, and bevacizumab, a regimen that yields good overall response rate and excellent overall survival and progression-free survival as a first-line treatment. The patient was then switched to second-line vinorelbine, gemcitabine, and bevacizumab combination for one month. During this time NGS testing reported ALK-positive mutation, and the patient progressed. Thus, the patient was switched to crizotinib, a targeted therapy effective for patients with ALK-positive mutations expected to prolong progression-free survival for about seven months, and during this time received whole brain radiation. She eventually progressed on crizotinib and eventually started on a clinical trial. She remained on the trial for twelve months until her death.
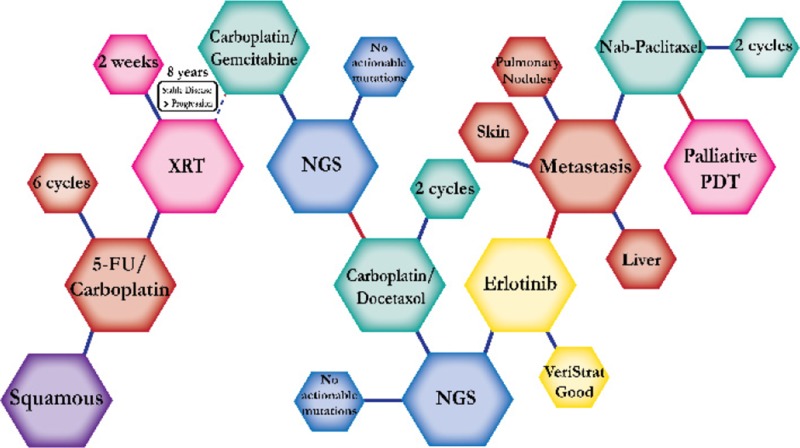	A 67 year-old Caucasian male smoker with history of esophageal cancer was first diagnosed with squamous cell carcinoma of the lung. He underwent adjuvant 5-FU and carboplatin chemoradiation. He did well for eight years until follow up workup confirmed metabolic activity. NGS testing reported no actionable mutations and the patient started on carboplatin and gemcitabine, a regimen found to have significantly higher efficacy in overall survival versus gemcitabine alone. He tolerated it well until disease progression, at which point the patient was switched to carboplatin and docetaxel, shown to have significant clinical benefit in progression-free survival. Another NGS test showed no actionable mutations and instead VeriStrat testing, a predictive prognostic response to erlotinib, was done and was deemed beneficial. Therefore, the patient started on erlotinib. Once he progressed, the patient was switched to nab-paclitaxel, but continued to progress. PDT was issued instead of nab-paclitaxel but there was little disease response and the patient died within four months. Photodynamic therapy uses photosensitizing agent along with light to kill cancer cells for a palliative effect in advanced NSCLC.
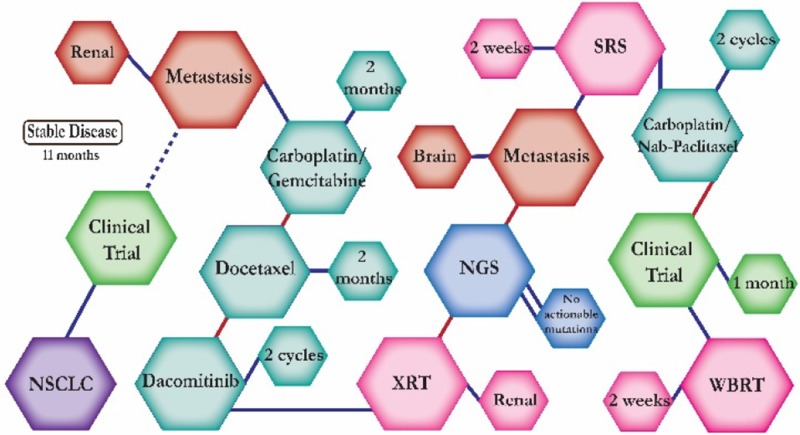	A 68 year-old African American male smoker who was first diagnosed with squamous cell carcinoma and was placed into a clinical trial. He had no progression for eleven months but then a biopsy showed metastatic disease and he was started on carboplatin and gemcitabine, a regimen found to have significantly higher efficacy in overall survival versus gemcitabine alone. Once he progressed, he was switched to docetaxel, a chemotherapy with significantly high 1-year survival rates as a second-line treatment, then dacomitinib but continued to progress. A NGS test revealed no actionable mutations. He was supposed to start on a clinical trial but was switched to SRS and chemotherapy due to progression of disease. Carboplatin and nab-paclitaxel was tolerated well but a follow up scan showed progressive disease. He started on a clinical trial but continued to be extremely symptomatic and even with whole brain radiation his condition worsened and there was little to no disease response. The patient died a month later.

The clinical structures of cases are read from left to right in chronological order.

## DISCUSSION

### Clinical heuristic framework

The genomic landscape of lung adenocarcinoma has not only advanced with the advent of next-generation sequencing, but also with the development of targetable therapeutics. The above 20 cases embody the overall multitude and complexity of cases an oncologist encounters throughout their career. The chemical structures of the patient treatment history were developed to understand the linear progression of each unique case as well as create a heuristic structure where the complexity of the structures indicated the complexity of decision-making. A heuristic, as defined by Gigerenzer and Gaissmaier, is a “strategy that ignores part of the information, with the goal of making decisions more quickly, frugally, and/or accurately than more complex methods” [10]. This methodology of thinking essentially describes the everyday rule-of-thumb that oncologists constantly use to leverage value-based medicine alongside evidence-based medicine to arrive at an optimal course of treatment for a patient. A physician relies on their own clinical expertise, intuition and past experience to quickly sift through a patient's clinical information and arrive at a decision that will be most beneficial to the patient. In most cases, the decisions made regarding treatment plans for patients are formulated from different sources: individual clinician insight, collaboration with other physicians, evidence-based clinical data, genomic testing, and more. The cases presented above show the divergence of clinical decision-making based on the initial conditions of each case. Though some cases may exhibit similar initial properties, such as the histology, metastatic disease, or an actionable mutation, each case diverges in functional outcomes based on the patient's progression and response.

The existence of a myriad of targets, both novel and traditional treatment options, has resulted in information overload with bewildering complexity in clinical decision-making. In addition, the advances in genomics and targeted therapies have not been accompanied with advances in the science of medical decision-making. A typical approach to improve practitioners’ decision-making is to develop evidence-based clinical practice guidelines (CPGs) by panels of experts who use their best clinical judgments to derive recommendations for practice. Increased explicitness and transparency in the process can be achieved by implementing CPGs as clinical pathways (CPs) (also known as clinical algorithms or flow-charts)[11]. There have been many frameworks, such as NCCN guidelines and pathways, that have attempted to streamline the process of clinical decision-making in oncology, but those methods have been limited by their inability to anticipate heterogeneity between individual patients. Expert decisions and situations in an uncertain world do not rely on estimating probabilities alone, but also on search rules, aspiration levels, lexicographic rules, and other heuristic principles [12]. However, we propose that it is possible to develop a platform of clinical decision-making that relies on categorical information to arrive at the most beneficial treatment option for the patient utilizing heuristics known as fast-and-frugal trees.

Fast-and-frugal trees can be best described as decision-making heuristics that can be analytically shown to provide the best possible solution to a problem with limited information [13]. They are typically presented as sequential “steps” taken with simple yes/no options that dictate a pathway in a decision tree tailored to the patient. Here we propose to establish a standardized template of fast-and-frugal trees for lung cancer decision-making that relies not only on established clinical expertise but also integrates value-based decision-making and published data into a heuristic platform. This would later be correlated and quantitated utilizing a large cohort of lung cancer patients in an effort to consolidate expertise in a quantitative manner (Figure 3). This “rule-of-thumb” decision-making to reach the best possible treatment would need to be verified and standardized in a quick manner to maximize benefit to patients. With the approach of more inhibitors, it will be essential to not only consider the genomic information but to correlate it with vital clinical information available with precision medicine. This is where heuristic strategies become significant by employing learned and evolved core capacities such as memory and recall to produce intuitive probabilities that are quick and rational [14]. Fast-and-frugal trees can be created and visualized utilizing the FFTrees package available in R [15]. The FFTrees function utilizes a training dataset as an argument, and generates several fast-and-frugal trees that attempt to classify patients into yes or no categories based on clinical information such as mutations and histology. The instructions for the package detail the step-by-step process for creating fast-and-frugal trees [15]. We believe this will be useful for decision-making, especially in the context of pathways that are being developed [16], so that all oncologists are able to arrive at these fast-and-frugal trees for clinical practice.

As an example, in straightforward case #2 we see a patient who was staged as pT3N1Mx, which is usually not considered for next-generation sequencing. However, a requested NGS test was able to identify an EGFR L858R mutation that then responded to erlotinib. The patient then progressed but was given combination therapy of erlotinib and afatinib to which she responded with stable disease. The benefit from NGS testing and combination treatment in this case outweighed the financial cost to the patient with a positive clinical outcome [17, 18]. This new work flow of ordering EGFR and ALK testing de novo radically transforms the treatment pathways a patient can undergo [19]. However, the detection of oncogenic mutations is not always clear cut as seen in complex case #1 where a patient who was ALK-negative by FISH but was determined to be ALK-positive by NGS [20]. The patient responded well to targeted ALK therapies, all because the second opinion testing by NGS transformed the treatment plan. This highlights both the negative and the positive value of genomic testing in that the FISH test had failed to determine an actionable mutation while the NGS test was able to detect it which offered the patient other treatment options. In this situation, heuristic of intuition was used to predict that the ALK FISH test may be inaccurate and an NGS test was ordered.

It is also important to note that though the list of targeted therapies for lung cancer continue to grow, there is still a gap between the mutational profile of all lung cancer patients and available therapeutics. For example, in straightforward case #3, we identified a patient whose genetic testing did not reveal any actionable mutations, but did reveal an R1068* mutation in EGFR. Heuristically from personal experience, it was predicted that a patient with a mutation in EGFR may benefit from TKI treatment, and so a VeriStrat**™** test, a predictive prognostic response to erlotinib, was ordered to determine if the patient would be eligible for TKI treatment [21]. Due to the VeriStrat**™** results, the patient was given and responded to erlotinib with minimal symptoms, highlighting the necessity of doubt and intuition when interpreting genomic mutations that do not at first appear to be actionable but can yield positive results when re-evaluated. However, while the VeriStrat**™** test results have sound prognostic value, they are not infallible as seen in complex case #9. Although the patient was reported to be VeriStrat**™** Good, erlotinib had little to no effect and ultimately resulted in progression of disease. On the other hand, there are more severe cases where the genetic testing does not offer the patient any options for treatment. For example, in straightforward case #4, the patient had no actionable genetic mutations and was offered chemotherapy but rapidly progressed before she could start treatment. In this case, the patient, who had a family history of lung cancer, was moved to hospice on the patient's wishes. This gives insight into the reality that rational decision-making in the clinic does not entirely rely on the highest expected utility, but must take into account the patient's values to avoid irrational overtreatment [22]. Unfortunately, cases with no actionable mutations are relatively frequent as seen in straightforward cases #6 and #9. Although both of thee patients were found to have a substitution mutation in KRAS, a driver mutation that comprises roughly 25% of all adenocarcinoma cases, it has no targetable therapy [23]. This reveals the level of uncertainty and intuitive thinking that is involved in oncology decision-making, especially considering there are currently no actionable treatments for a substantial portion of lung adenocarcinoma mutations.

Though the use of targeted therapies has revolutionized lung cancer treatment options, it is vital to remember that they are not always guaranteed for success and resistance to therapies often develops in most cases [24]. In the example of complex case #6, it is shown that the patient had developed progression on crizotinib and was switched to six cycles of cisplatin and pemetrexed. There was noted to be a repeat sensitivity to pemetrexed after a treatment holiday and the patient continued for another seven months with crizotinib and pemetrexed combination maintenance [25, 26]. The equation for clinical decision-making becomes more complex when considering the mechanisms of resistance to targeted inhibitors. This is observed in complex case #5 where the patient was able to achieve prolonged survival on three different ALK inhibitors by switching between ALK inhibitors and cytotoxic chemotherapy to circumvent progression and tumor resistance [27]. This treatment plan of switching off between different tyrosine kinases inhibitors or between different TKIs and cytotoxic chemotherapy is noted in other cases as well [25, 28, 29]. These multiple cases reveal potential treatment pathways for patients who initially benefitted from crizotinib or erlotinib yet eventually progressed—but the majority of these treatment decisions were done based on intuition and quick re-evaluation of published evidence rather than definitive linear treatment pathways. This highlights the restrictions of evidence-based medicine where physicians are forced to follow guided paths with unsubstantiated linear outcomes rather than inference from patient value, tacit knowledge, and problem-solving frameworks [30]. The need for a physician-focused method of evaluating treatment options is essential as shown in Figure 4, especially as the landscape of targeted therapies continues to expand.

It is current NCCN guidelines to obtain molecular information on patients with adenocarcinoma variant of non-small cell lung cancer. To obtain this information, there are several vendors in this space including broad genomic panels (FoundationOne, Guardant 360) or single mutation analysis (e.g. BIOCEPT). However, sequencing of therapies has become more complex. Because the genomics of NSCLC can change over time and by site of metastatic disease, it is likely that the temporal integration of multiple liquid biopsy time points will be needed in the clinic to optimize treatment choice. As with any other test, the genomic testing can be noninformative or the interpretation can be challenging, as was illustrated in several cases described above. Temporal changes in genomics will be easier to interpret with longitudinal tracking software that can be accessed by the oncologist. However, we suggest that healthcare, oncology care especially, can be improved by the combination of a heuristic cognitive architecture, driven by human intuition and emotion, and a structured computational framework that may provide statistically viable approximations that can mimic everyday expertise in dealing with clinical uncertainty to arrive at a model of rationality that integrates patient values with treatment utility [22, 31]. Heuristic concepts are applied in certain branches of medicine, such as cardiovascular decision-making, specifically in the efficacy of fast-and-frugal trees in prescribing statins to prevent cardiovascular disease [32, 33]. This specialized FFT designed to assist in medical decision-making generated numerous permutations of pathways a physician could undergo depending on the patient criteria. This strategy was also applied in predicting the causes of community-acquired pneumonia in young children so that the appropriate antibiotic treatment is prescribed [34]. This study is a first analysis determining the decision-making value in the context of lung cancer and molecular analysis. It will be important to determine the value of other mutations besides EGFR and ALK through further analysis of other mutations such as ROS1, MET, HER2, BRAF, TRK, and others. We hope this forms a nidus for the future for further studies and robust dataset with large number of patients.

The field of genomics in NSCLC has exponentially grown within the last decade alone. The introduction of molecular testing and NGS have radically shifted the era of standardized healthcare in NSCLC to a more personalized and precise one as it continues to advance [35]. We witness the rapid changes regarding the value and efficacy of targeted therapies in different cases as seen by the recent FDA approval of immunotherapy pembrolizumab with carboplatin and pemetrexed as a first-line treatment in metastatic NSCLC [36, 37]. Even more recent, pembrolizumab was approved by the FDA as the first drug universally used based on a genetic biomarker of microsatellite instability-high or mismatch repair deficient instead of by cancer type [38]. This marks the first approval of a drug not manufactured for a particular type of cancer but instead by biomarkers expression in tumor cells regardless of its origin. This blurs the lines we initially placed between cancer types and introduces a new drug classification and potential research focuses based on tumor biomarkers. The value of genomic testing and personalized treatment plans is necessary as the landscape of NSCLC continues to evolve.

In much of oncology, the physician follows a well laid path, e.g. an NCCN guideline, or mimics a clinical trial that applies to the patient's clinical situation. However, as illustrated in these cases, many patients do not fall into a simple, uniform pathway on the NCCN guidelines so the oncologist must make decisions based on imprecise information through observing the results and discernment. Since there is no one “standard of care” in the new era of treating lung cancer, clinicians tend to find themselves on precarious grounds. The ability of the clinical oncologist to keep up with changes scientifically is being taxed as pathways are constantly evolving due to the introduction of newer medicine and treatment. Integrating disparate sources of knowledge becomes more complex and the physician lacks “point of care epidemiology” tools. Physicians in clinic must input multiple Bayesian priors—from history, physical examination, the medical literature, and our past successes and failures—to derive a treatment plan for each individual patient. The clinician is tasked with doing this in real time in a field such as NSCLC where the science is increasing at a rapid pace. We are at a crossroads where the complexity of managing patients with NSCLC is increasing at a rate that oncologists face time constraints [39]. This is the time when medical informatics, specifically heuristic decision-making and fast-and-frugal trees, can be judiciously applied to improve patient outcomes in NSCLC.

## MATERIALS AND METHODS

### Patients and subjects

Lung adenocarcinoma patients included in this analysis were evaluated at the University of Chicago Hospitals (UCH) from 2008 to 2015 and underwent genotype testing as requested by the primary clinical provider (n=20). Patients were consented to the Institutional Review Board (IRB) approved protocols 9571 and 13473, allowing our research team to obtain detailed clinical information such as course of treatment as well as the results of the clinical genetic testing to be analyzed. The testing was done through next-generation sequencing (NGS) and all data was reported in compliance with the Health Insurance Portability and Accountability Act (HIPAA) regulations.

### Study design

Clinical patient data was collected, after consent, from both the UCH electronic medical records (EPIC) medical database and the Thoracic Oncology Research Program (TORP) Microsoft Access Databases. This allowed the research team to collect demographic data such as age, sex, race, histology, treatment course, and functional outcomes. In addition to the clinical data in the hospital database, tumor tissue testing results were obtained from a NGS provider.

### Statistical analysis

No formal statistical hypotheses were assessed and all statistical analyses were descriptive. The sample size was determined by examining ten straightforward and ten complex cases that each had unique clinical insight.

### Methods

The patient timelines and fast-and-frugal trees were created using Adobe Illustrator to display the unique features and differences of individual cases. The mutational tile plot was created by coding the type of mutation reported to a number in an excel spreadsheet. The spreadsheet was then uploaded into R programming and modified using the publicly available “pheatmap” module.

## SUPPLEMENTARY MATERIALS FIGURES



## Abbreviations

NSCLC: Non-small cell lung cancer
NCCN: National Comprehensive Cancer Network
ALK: Anaplastic lymphoma kinase
EGFR: Epidermal growth factor receptor
IRB: Institutional Review Board
UCH: University of Chicago Hospital
TORP: Thoracic Oncology Research Program
FISH: Fluorescent in situ hybridization
NGS: Next-Generation Sequencing
TKI: Tyrosine kinase inhibitors
FDA: U.S. Food and Drug Administration
