# Supplementary material for: Heuristic value-based framework for lung cancer decision-making
Source: Oncotarget. 2018 Jul 6;9(52):29877–91. doi: 10.18632/oncotarget.25643 (PMC6057456; doi:10.18632/oncotarget.25643)
Supplement: Supplementary file 1 [file oncotarget-09-29877-s001.pdf]

# Heuristic value-based framework for lung cancer decision-making

## SUPPLEMENTARY MATERIALS

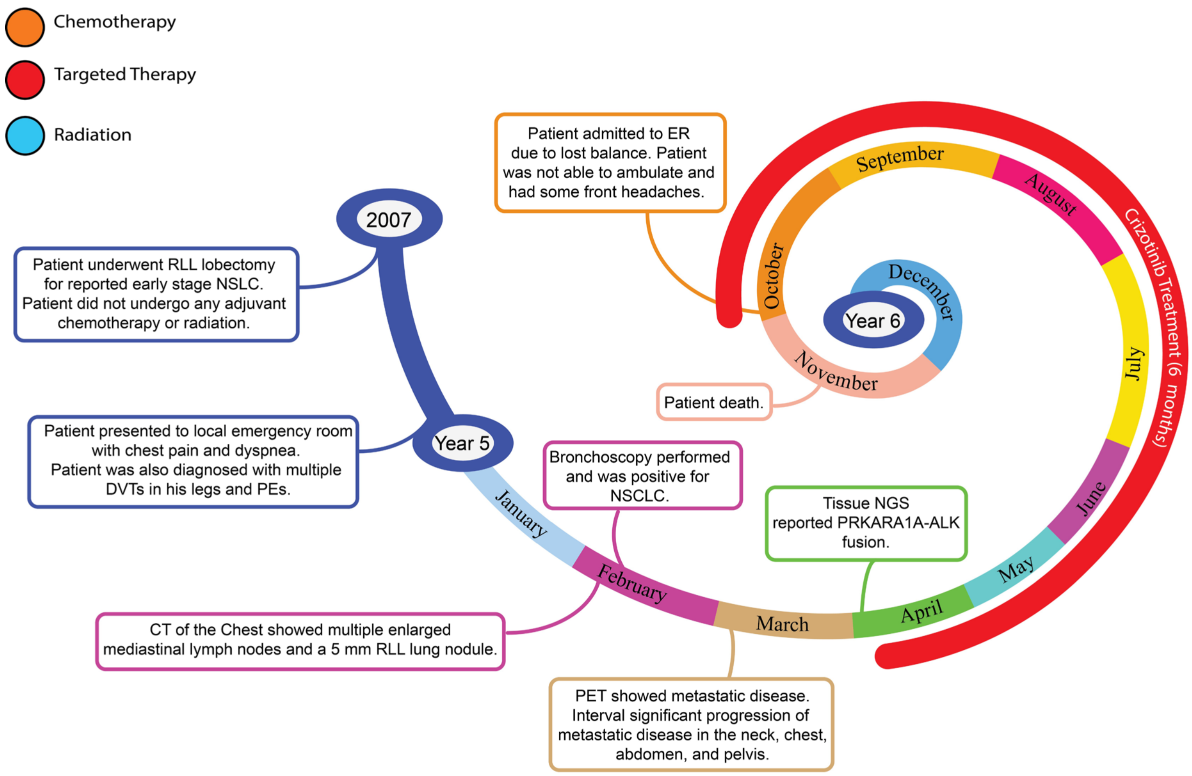


**Supplementary Figure 1: Straightforward and Complex timelines of 20 patients.**

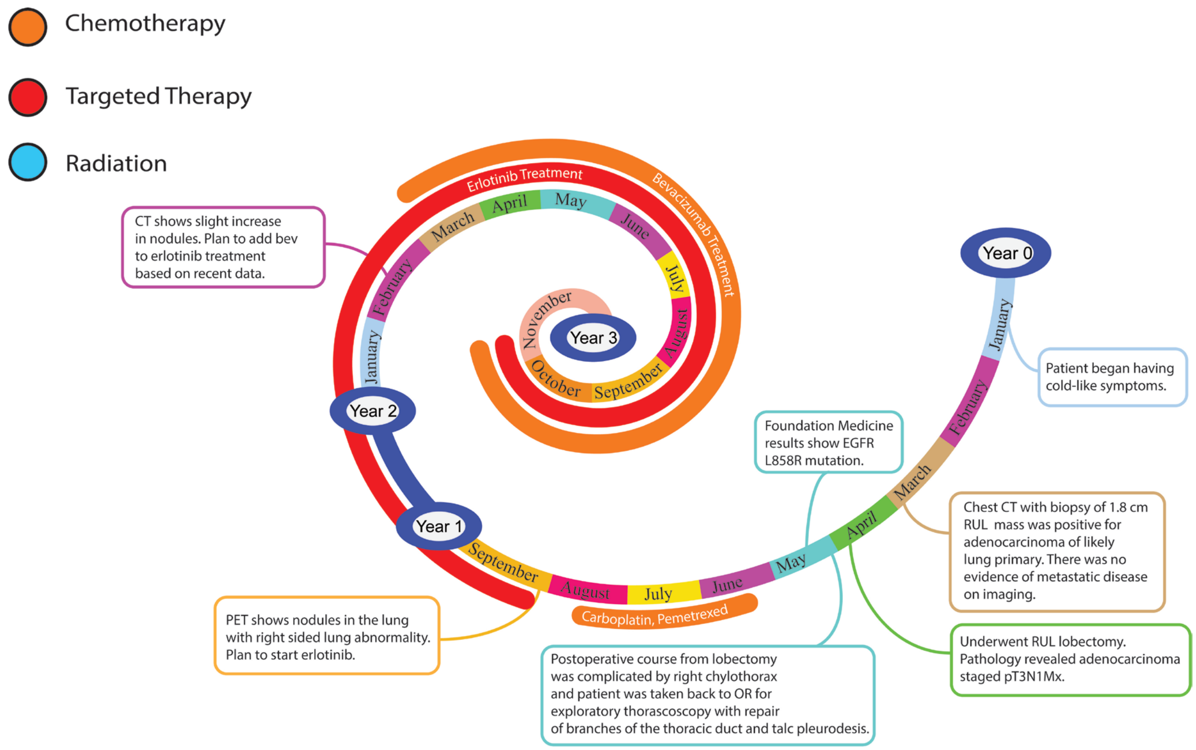


**Supplementary Figure 2: Straightforward and Complex timelines of 20 patients.**

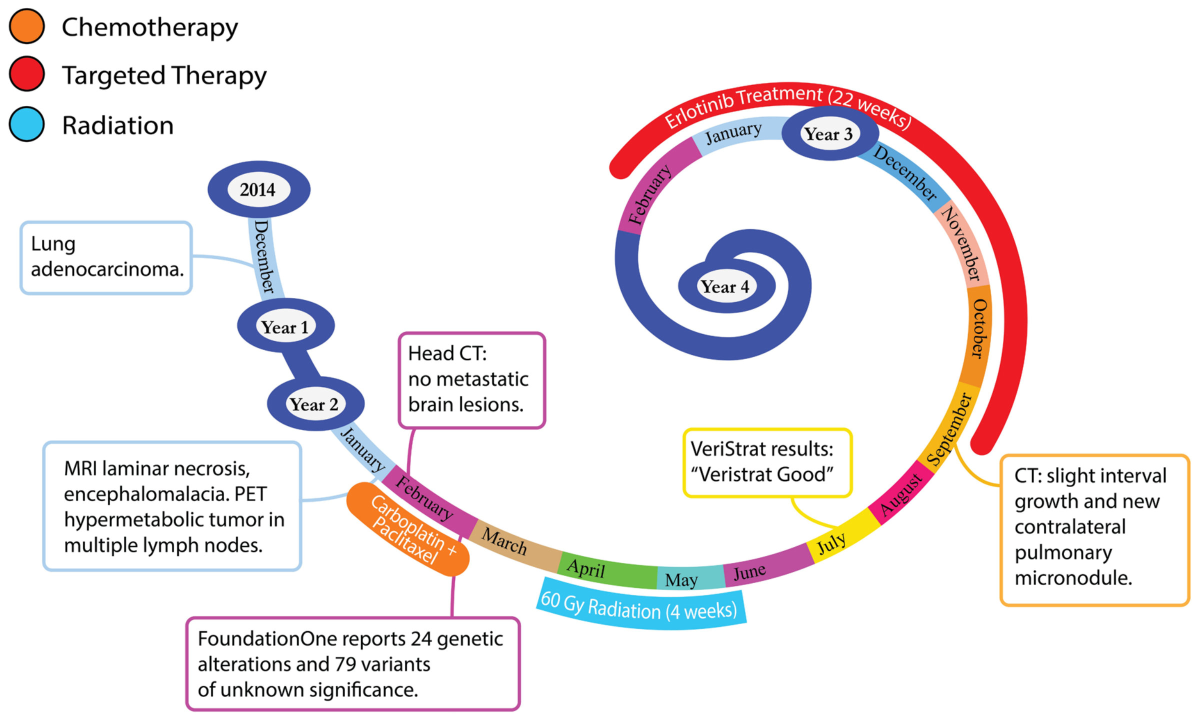


**Supplementary Figure 3: Straightforward and Complex timelines of 20 patients.**

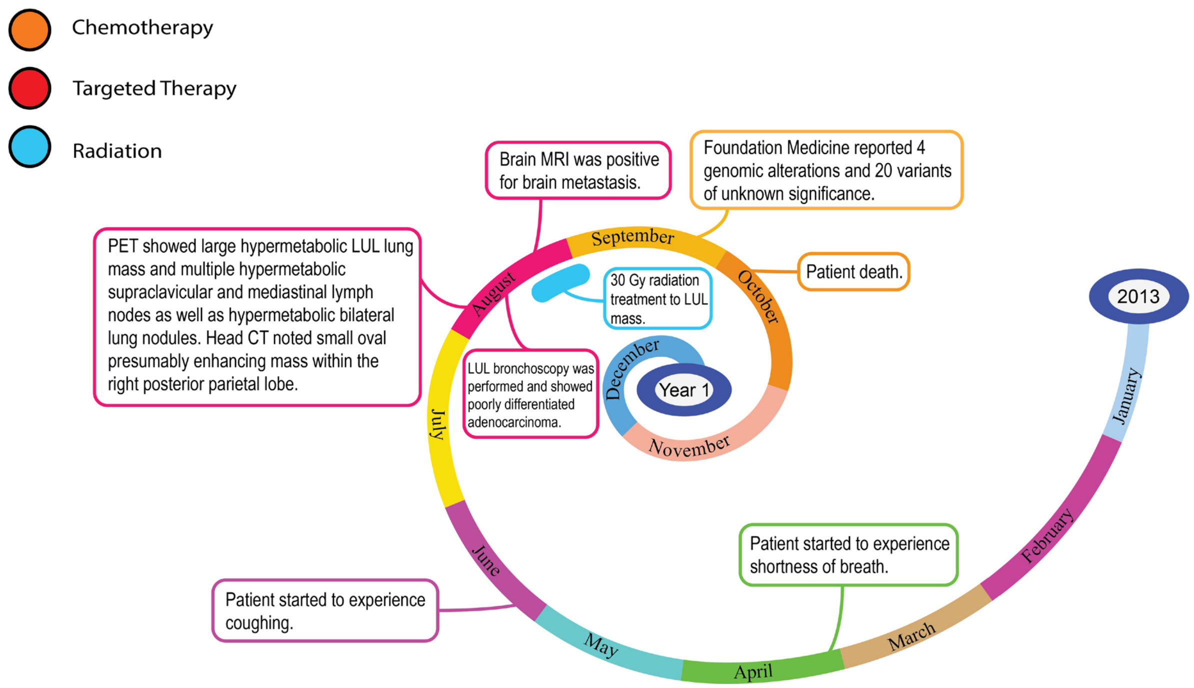


**Supplementary Figure 4: Straightforward and Complex timelines of 20 patients.**

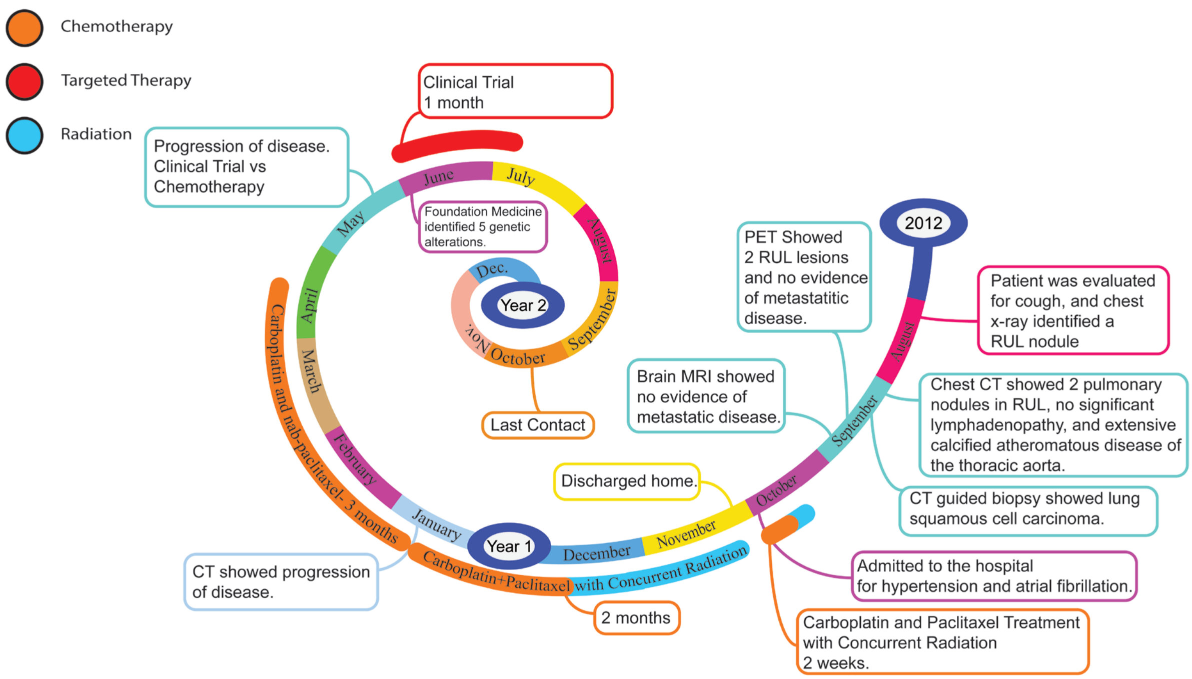


**Supplementary Figure 5: Straightforward and Complex timelines of 20 patients.**

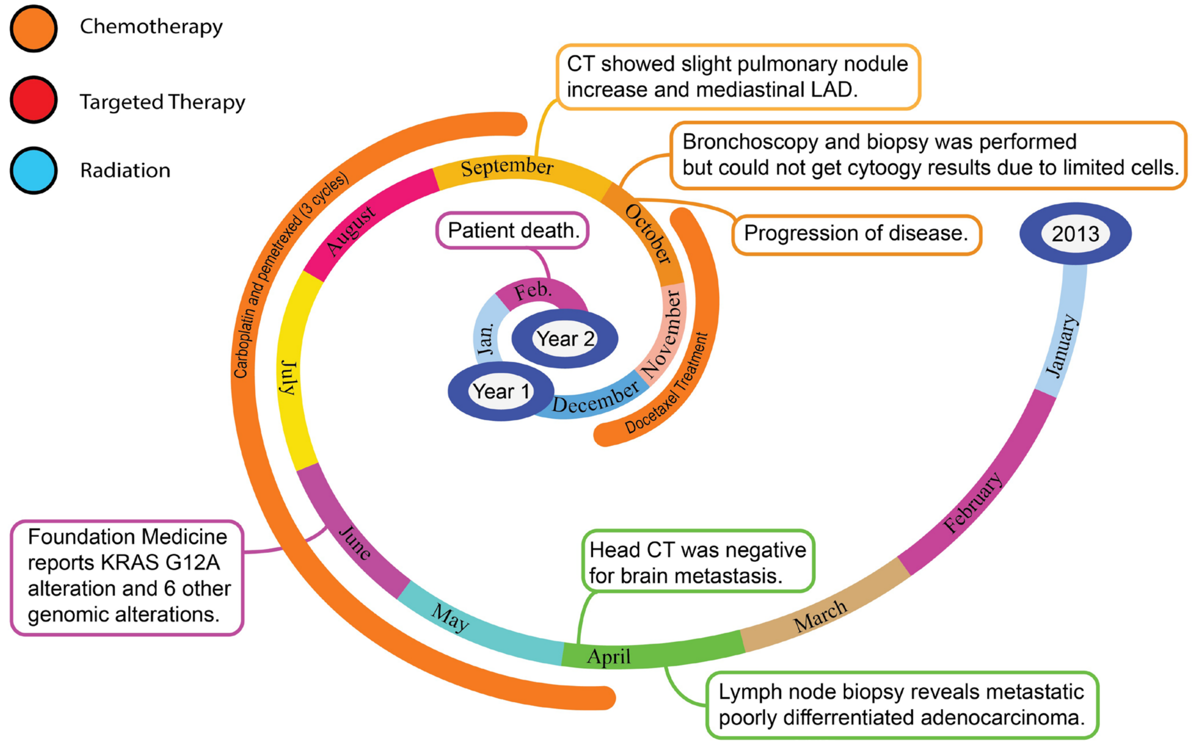


**Supplementary Figure 6: Straightforward and Complex timelines of 20 patients.**

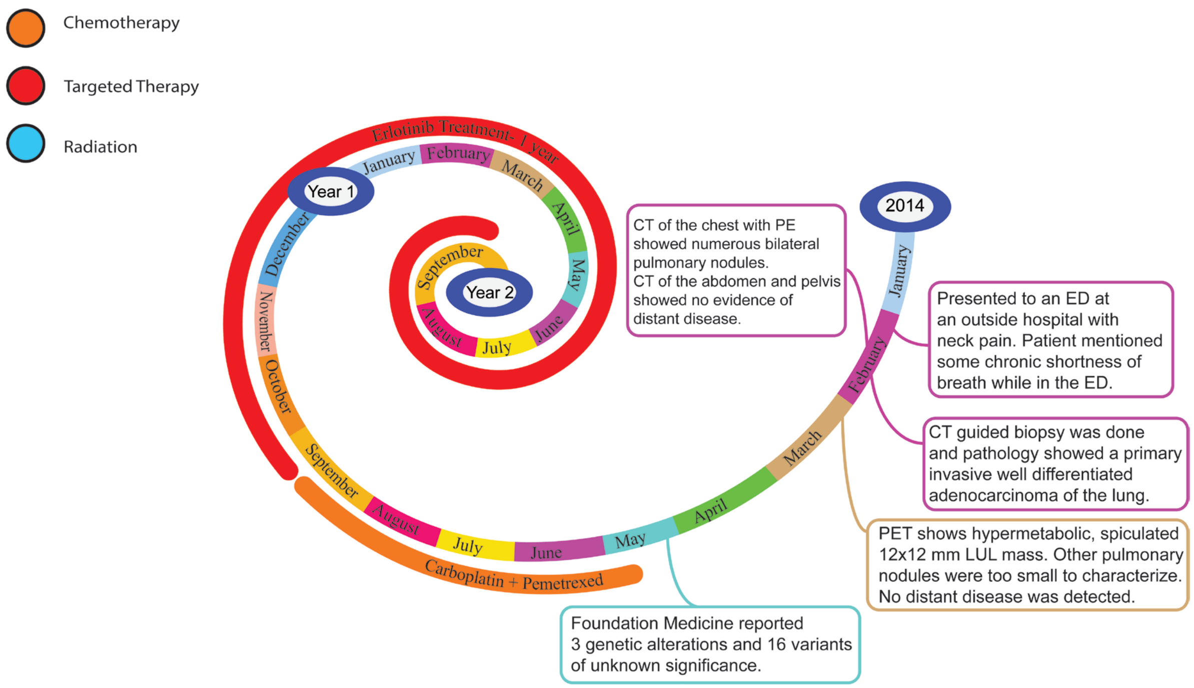


**Supplementary Figure 7: Straightforward and Complex timelines of 20 patients.**

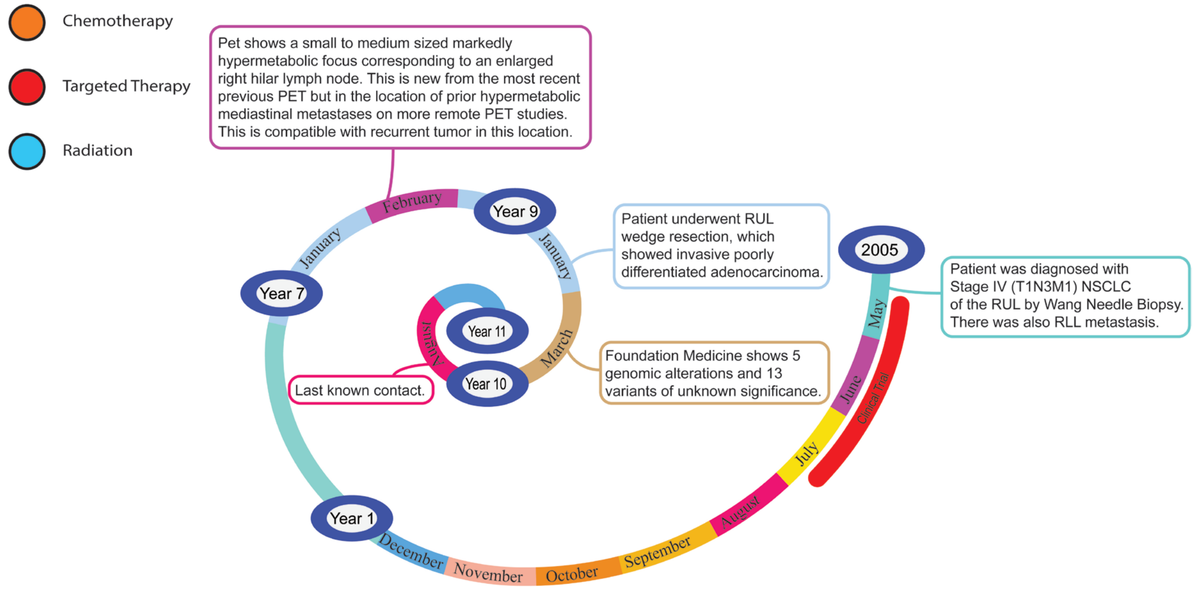


**Supplementary Figure 8: Straightforward and Complex timelines of 20 patients.**

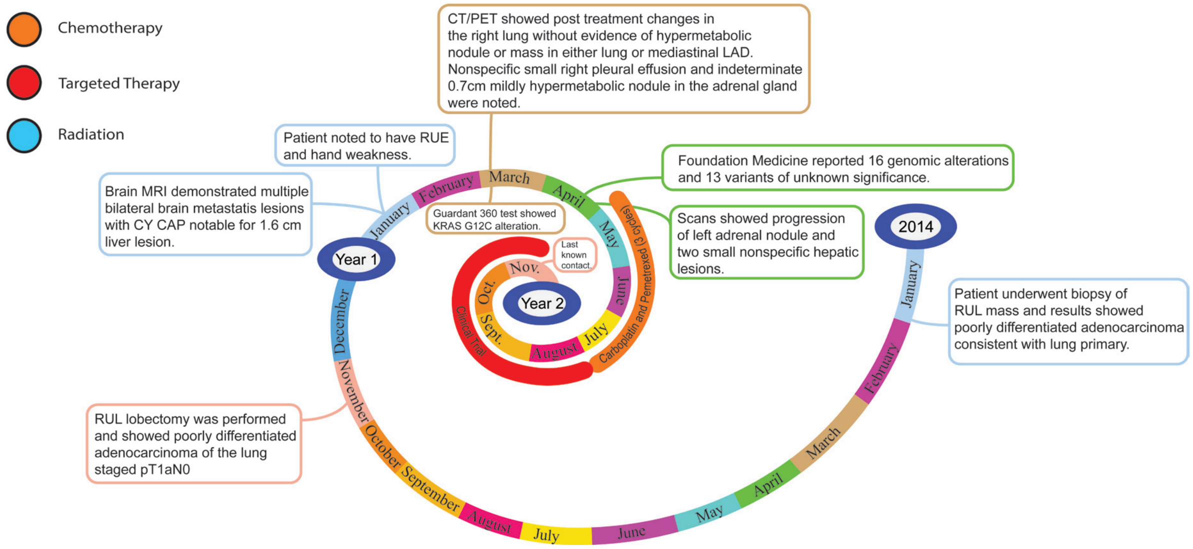


**Supplementary Figure 9: Straightforward and Complex timelines of 20 patients.**

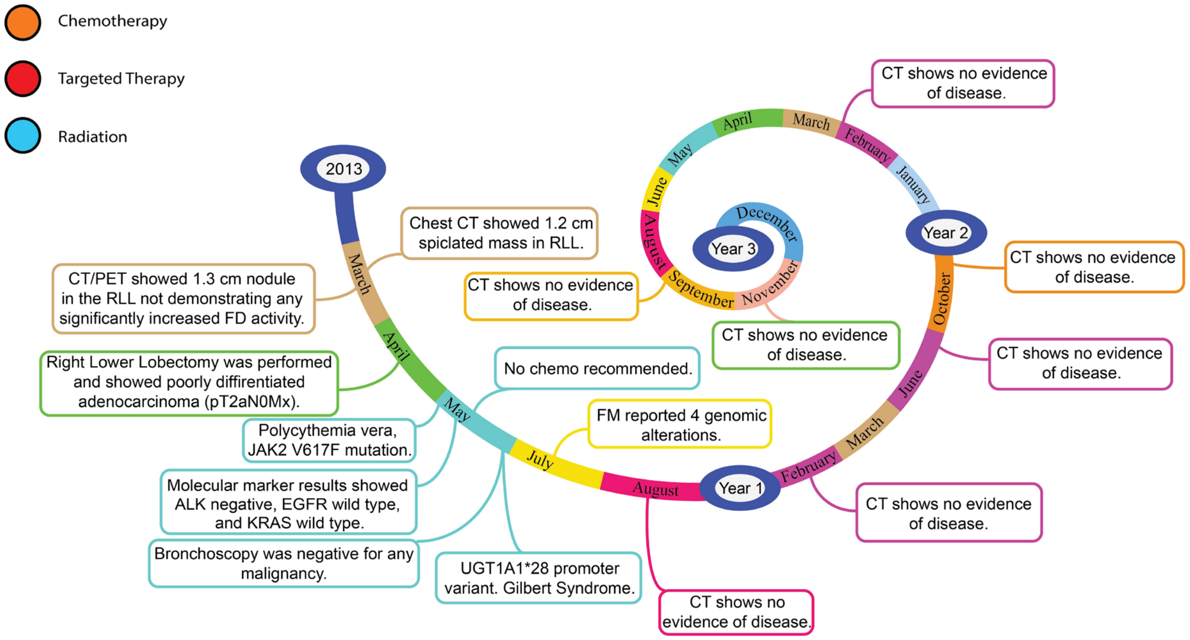


**Supplementary Figure 10: Straightforward and Complex timelines of 20 patients.**

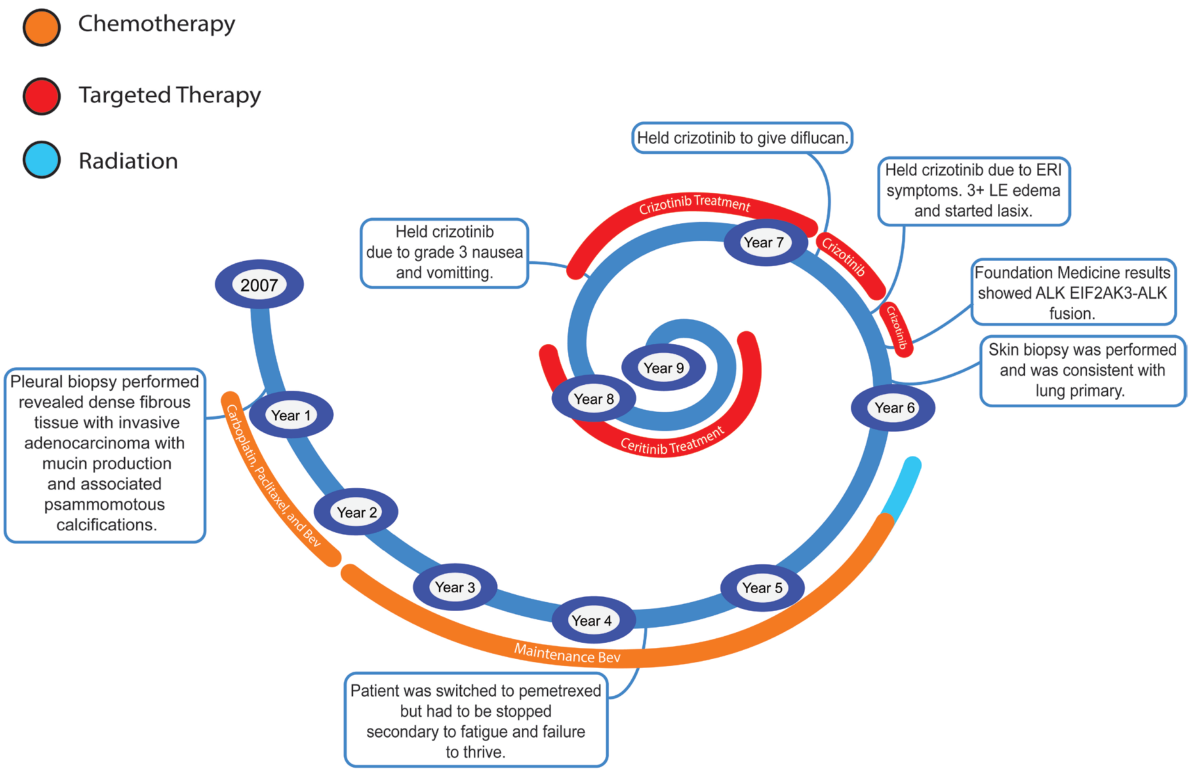


**Supplementary Figure 11: Straightforward and Complex timelines of 20 patients.**

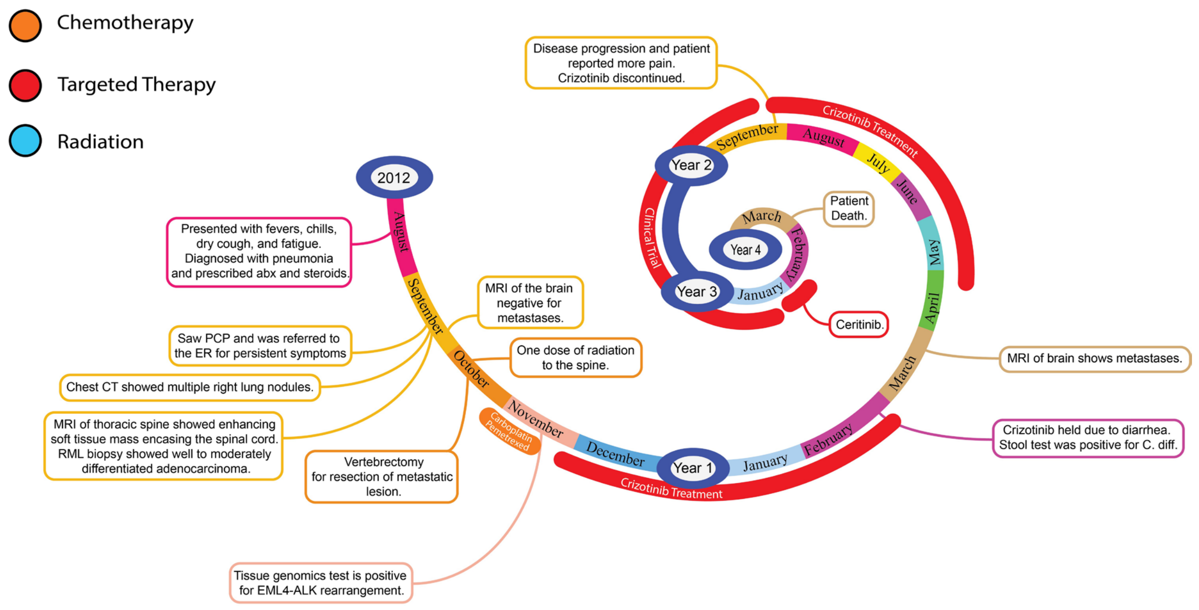


**Supplementary Figure 12: Straightforward and Complex timelines of 20 patients.**

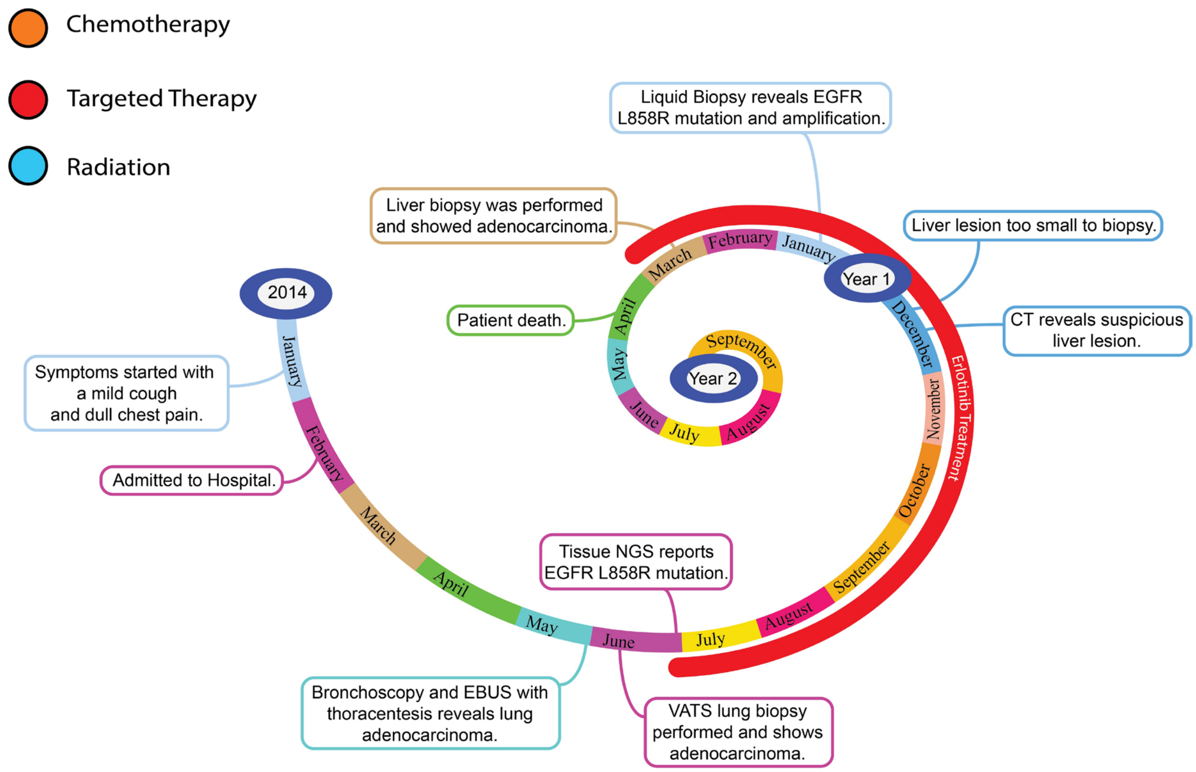


**Supplementary Figure 13: Straightforward and Complex timelines of 20 patients.**

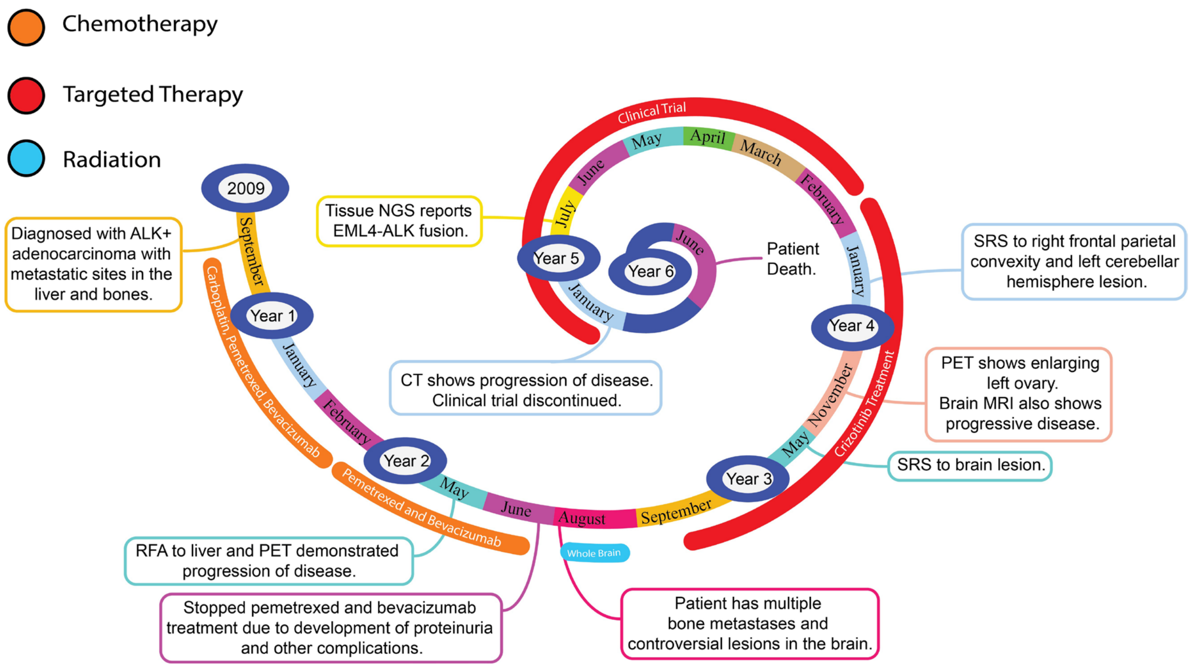


**Supplementary Figure 14: Straightforward and Complex timelines of 20 patients.**

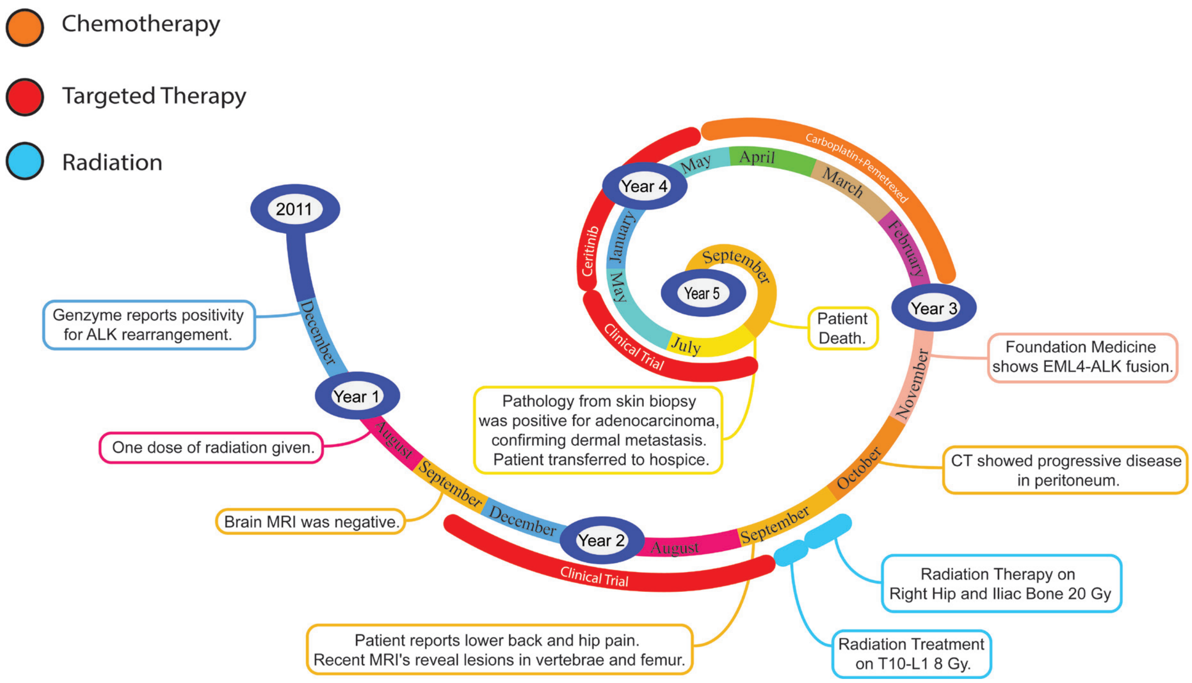


**Supplementary Figure 15: Straightforward and Complex timelines of 20 patients.**

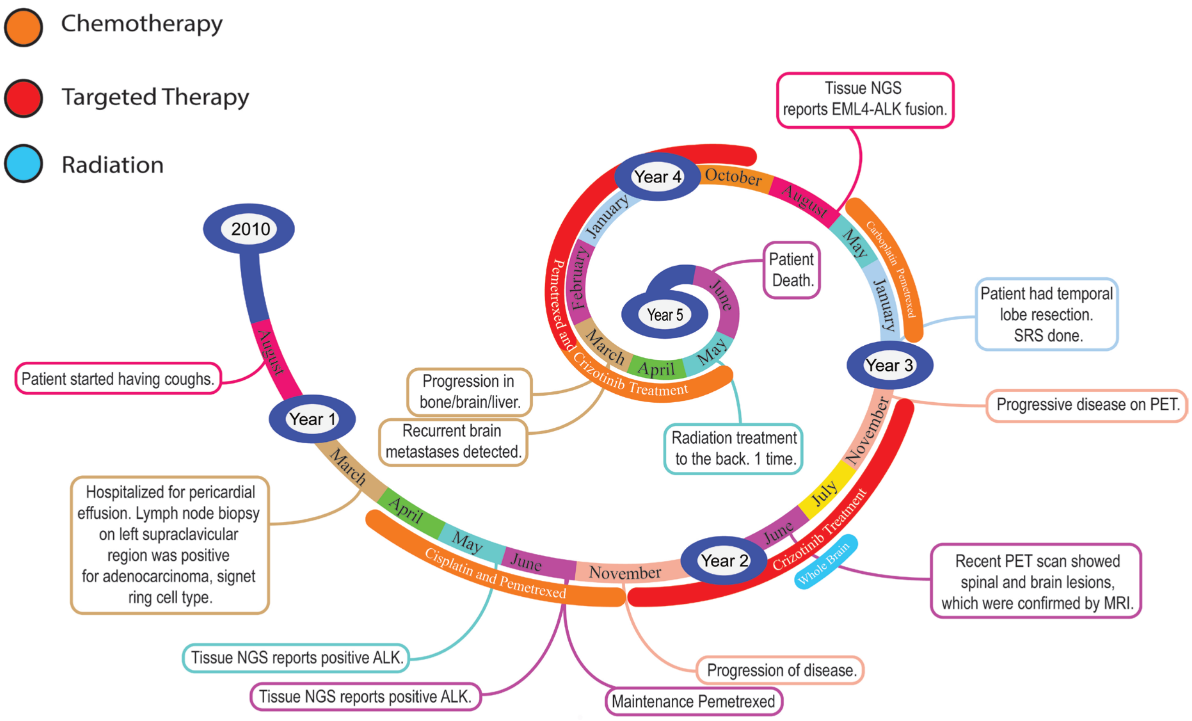


**Supplementary Figure 16: Straightforward and Complex timelines of 20 patients.**

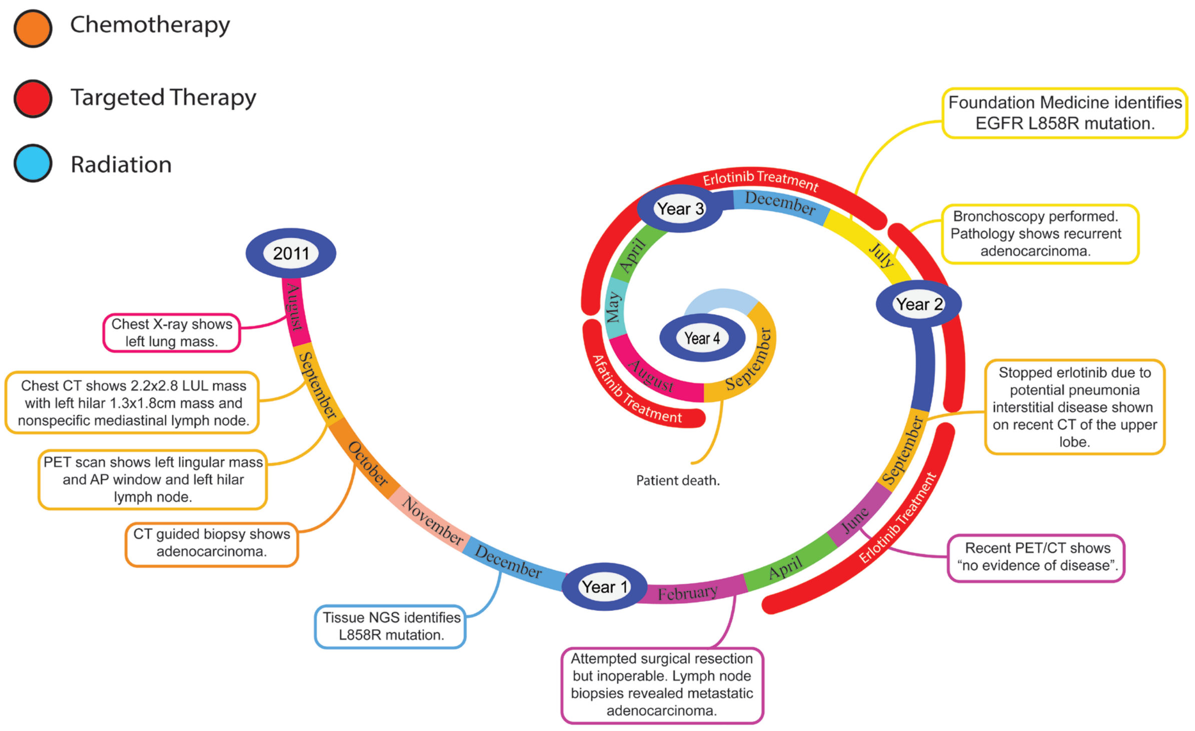


**Supplementary Figure 17: Straightforward and Complex timelines of 20 patients.**

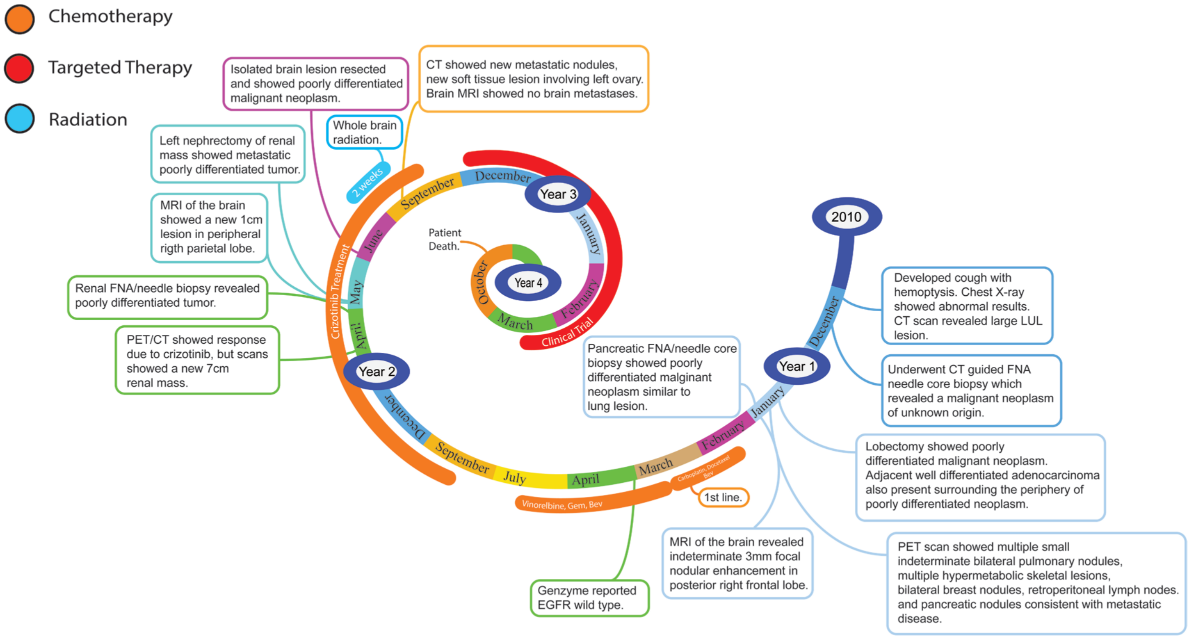


**Supplementary Figure 18: Straightforward and Complex timelines of 20 patients.**

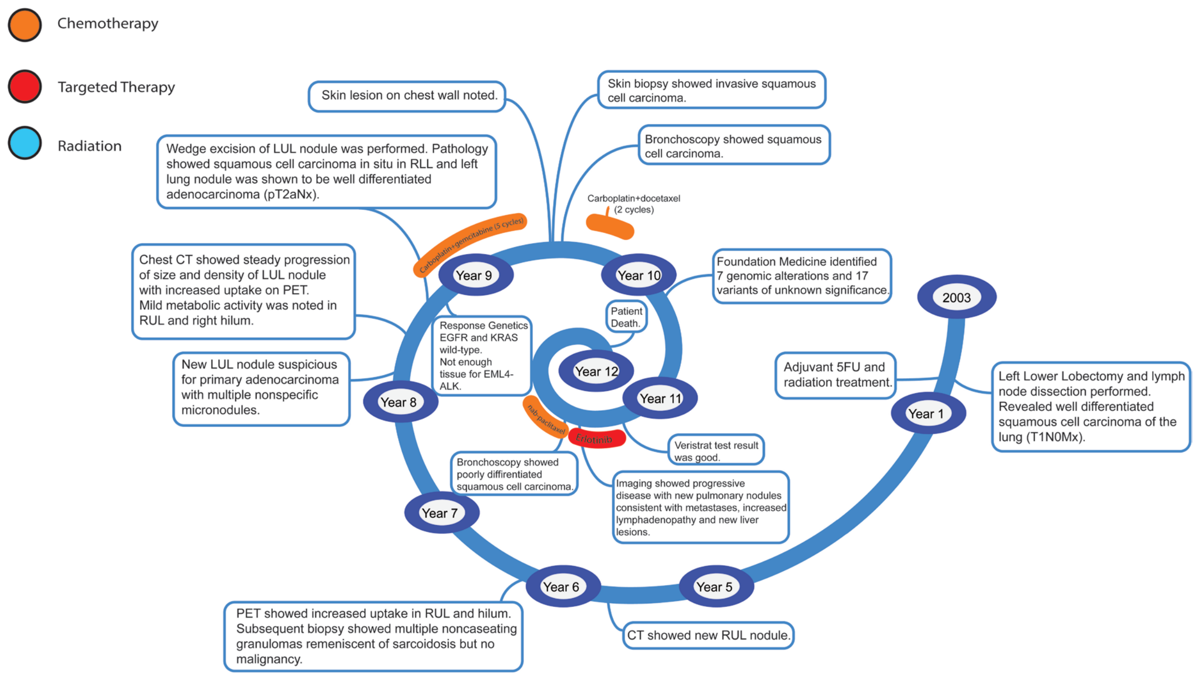


**Supplementary Figure 19: Straightforward and Complex timelines of 20 patients.**

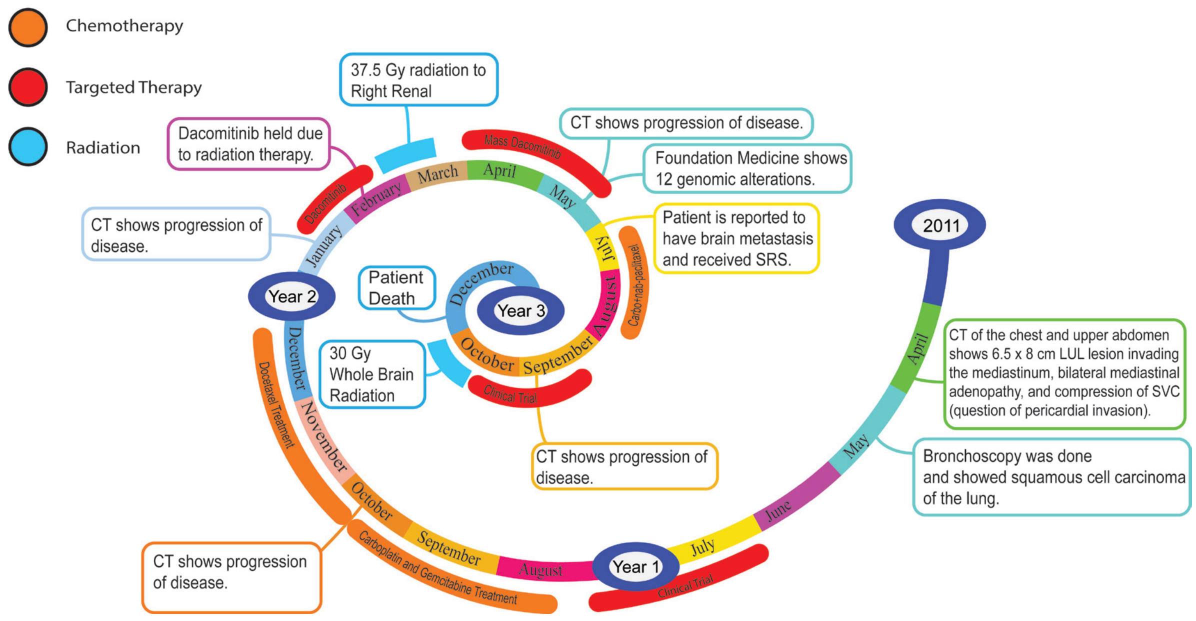


**Supplementary Figure 20: Straightforward and Complex timelines of 20 patients.**
